# Designing Metal Phosphide Solid-Electrolyte Interphase for Stable Lithium Metal Batteries Through Electrified Interface Optimization and Synergistic Conversion

**DOI:** 10.1007/s40820-025-01813-1

**Published:** 2025-06-27

**Authors:** Jung Been Park, Changhoon Choi, Min Sang Kim, Hyeongbeom Kang, Eunji Kwon, Seungho Yu, Dong-Wan Kim

**Affiliations:** 1 School of Civil, Environmental, and Architectural Engineering, Korea University, Seoul, 02841 Republic of Korea; 2 Department of Materials Science and Engineering, SungShin Women’s University, Seoul, 01133 Republic of Korea; 3 Energy Storage Research Center, Korea Institute of Science and Technology, 5, Hwarang-ro 14-gil, Seongbuk-gu, Seoul, 02792 Republic of Korea; 4 Division of Energy and Environment Technology, KIST School, Korea University of Science and Technology, Seoul, 02792 Republic of Korea

**Keywords:** Li metal batteries, Heterostructures, In situ reactions, Dendrite-free anodes, Mixed ionic/electronic conductors

## Abstract

Regulating the nucleation and growth of Li metal is crucial for achieving stable high-energy-density Li metal batteries (LMBs) without dendritic Li growth, severe volume expansion, and “dead Li” accumulation. Herein, we present a modulation layer composed of porous SnP_0.94_/CoP *p-n* heterojunction particles (SCP), synthesized applying the Kirkendall effect. The unique heterointerfaces in the SCP induce a fully ionized depletion region and built-in electric field. This provides strong Li affinity, additional adsorption sites, and facilitated electron transfer, thereby guiding dendrite-free Li nucleation/growth with a low Li deposition overpotential. Moreover, the strategic design of the SCP, accounting for its reaction with Li, yields electronically conductive Co, lithiophilic Li–Sn alloy, and ionic conductive Li_3_P during progressive cycles. The mixed electronic and ionic conductor (MEIC) ensure the long-term stability of the SCP modulation layer. With this layer, the SCP@Li symmetric cell maintains a low overpotential for 750 cycles even at a high current density of 5 mA cm^−2^. Additionally, the LiFePO_4_//SCP@Li full cell achieves an imperceptible capacity decay of 0.03% per cycle for 800 cycles at 0.5 C. This study provides insight into MEIC heterostructures for high-performance LMBs.

**Supplementary Information:**

The online version contains supplementary material available at 10.1007/s40820-025-01813-1.

## Highlights

- Through strategic heterostructure design, it is possible to create electrified interfaces that induce the formation of a fully ionized depletion region and a built-in electric field.
- These electrified interfaces enhance Li affinity and promote uniform current distribution, leading to stable Li deposition behavior.
- Owing to intentional design, SnP_0.94_/CoP heterostructure enables in situ conversion to a mixed ionic/electronic conductor during cycling, ensuring the long lifespan.

**Supplementary Information:**

The online version contains supplementary material available at 10.1007/s40820-025-01813-1.

## Graphical abstract

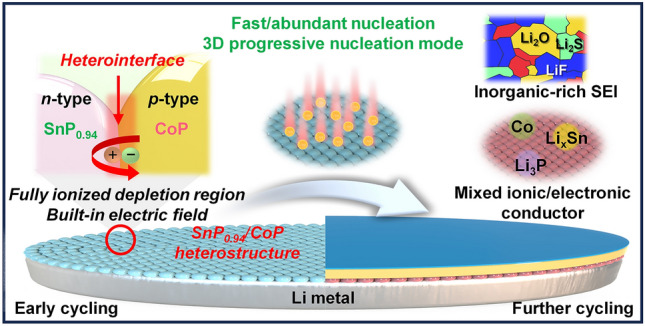

## Introduction

With the increase in global focus on environmental sustainability, secondary battery technology has advanced rapidly [1]. In addition, the growing demand for energy storage systems with higher energy densities (impelled by the rapid expansion of electric vehicles and consumer electronics markets) emphasizes the necessity of developing next-generation energy storage systems that surpass commercialized Li-ion batteries (LIBs) [2, 3]. Among the various candidates, lithium metal batteries (LMBs) constructed with Li metal anodes (LMAs) are widely considered as potential successors to LIBs for near-future energy markets because of their high theoretical capacity (3860 mAh g^−1^), low redox potential (− 3.04 V vs. standard hydrogen electrode (SHE)), and low gravimetric density (0.59 g cm^−3^) [4, 5]. Nonetheless, the practical utilization of LMBs is impeded by persistent technical issues. The “hostless” LMAs with high reactivity can result in non-uniform Li-ion flux and irregular Li tip formation, which intensify the local charge density and electric field (called “tip effect”) [6]. Because the generated Li tips tend to evolve into Li dendrites, LMAs typically undergo infinite volume expansion and substantial mechanical stress, leading to damage the naturally formed solid electrolyte interphase (SEI) and the formation of inactive “dead” Li [7]. This mainly contributes to the continual consumption of organic electrolytes, degradation of electrochemical performance (particularly, the Coulombic efficiency (CE) and capacity decay), and safety hazard [8, 9]. Therefore, innovative approaches to stabilizing LMAs against dendrite formation and undesirable side reactions are essential for high-performance and safe LMB fabrication.

In response to the aforementioned challenges, numerous effective approaches have been proposed to mitigate the rambling growth of Li dendrites and side reactions, such as, adjusting the electrolyte composition [10, 11], introducing a modified separator [12], designing of 3D host/current collector [13, 14], adopting durable solid-state electrolytes [15, 16] and interfacial engineering of Li surfaces. Fine-tuning the electrolyte composition by adding functional additives (e.g., LiNO_3_ [17], fluoroethylene carbonate [18], and heptafluorobutyrylimidazole [19]) can regulate the chemical composition and structure of the SEI. This ensures its relative stability in the short term. However, these additives are gradually consumed, resulting in the collapse of the SEI and its accumulation during the prolonged cycling of LMBs because of the high reactivity of Li [20]. Based on Sand’s time theory, 3D conductive host/current collectors with ample space, functioning as a “shelter” for LMAs, can effectively ensure reduced local current density and homogenized electric field/Li-ion flux distribution. Simultaneously, it can accommodate the volume expansion originating from the Li plating/stripping process [21, 22]. However, commonly studied 3D host and metal-based current collectors such as Cu and Ni foams tend to be excessively thick. These generally comprise over 83 wt% of the electrode [23]. Also, most require sophisticated synthesis techniques and pre-lithiation processes with limited Li storage capacity [24, 25]. Introducing solid-state electrolytes can improve the ion transportation efficiency, decrease the interfacial resistance, and increase the power output of LMBs [26, 27]. However, this approach typically entails a trade-off between mechanical strength and compatibility with LMAs [28]. The interfacial engineering directly on the LMA surface mainly involves (i) the construction of an artificial protective layer or SEI (inducing Li deposition below the artificial layer) and (ii) creating a modulation layer (inducing Li deposition above the modulation layer). This can effectively suppress dendrite growth and side reactions without compromising CEs during repeated Li plating/stripping processes. However, because of the insufficient electronic/ionic conductivity and mechanical stability of most reported protective layers and SEIs, these are typically effective only at relatively low current densities (< 3.0 mA cm^−2^) [29]. In this regard, as a potential alternative strategy to enable long-term repeated Li plating/stripping at practical current densities higher than 3.0 mA cm^−2^, the modulation layer on Li surfaces has recently attracted researchers’ attention. Preferably, to activate the anode electrolyte interface, the modulation layer should display a remarkable mixed electronic/ionic conductivity (MEIC) and high surface Li-ion affinity. First, owing to its high electronic/ionic conductivity, the modulation layer enables LMAs to enhance interfacial kinetics by reducing the concentration polarization at the interface through accelerated Li-ion dispersion and migration [30–32]. Second, the strong interfacial affinity for Li ions promotes uniform Li nucleation and increases the lateral diffusion of Li adatoms. This prevents the formation of 1D Li dendrites. Building on this synergistic effect, we can anticipate a preferable modulation layer to enable fast and uniform Li-ion flux distribution to provide adequate nucleation sites and induce lateral dendrite-free Li growth [33]. To realize these potential modulation layers, designing the chemical composition and microstructure of the materials is critically important.

Various lithiophilic inorganic materials have been proposed using diverse strategies for fabricating stable LMA. With respect to the most widely reported metal oxide/sulfides, although these lithiophilic species exhibiting strong interaction with Li ions can induce low nucleation overpotential and uniform Li nucleation/deposition of LMAs [34–36], inferior Li-ion conductive Li_2_O/Li_2_S is usually formed by a reaction between Li and the oxides/sulfides (0 > ΔG). It is detrimental to the distribution of local ions/electrons and uniform Li plating/stripping [37]. Meanwhile, lithiophilic metal phosphides (e.g., Cu_3_P [38, 39], Ni_3_P [40], and CoP [41]) have recently been regarded as a noteworthy alternative class with relatively good electrical conductivity and chemical integrity. These can ensure a homogeneous Li-ion flux by using higher Li-ion conductive Li_3_P (~10^−4^ S cm^−1^, compared with Li_2_O/Li_2_S) synthesized from the reaction between Li and the phosphides [42, 43]. It is widely acknowledged that the interfaces of heterogeneous materials with elaborately designed microstructures can play a crucial role in controlling the interfacial charge redistribution and Li-ion adsorption capacity [44]. When two materials (A and B) are introduced into a heterojunction structure, electrons are transported spontaneously from material A with a higher Fermi level (E_F_) to material B with a lower E_F_ until the E_F_ align. Depending on their band structures, these heterogeneous materials can form a fully ionized depletion region (FIDR). It provides abundant Li-ion adsorption sites and a built-in electric field (BIEF) that facilitates charge redistribution [45, 46]. Owing to the advantages of these heterointerfaces, previous studies reported the use of heterostructured materials for stable LMAs. However, many of these studies involved modifications to the 3D Li host or current collectors [47–49]. The modifications can cause the electrochemical or thermodynamic decomposition of the materials during the pre-lithiation process, thereby obscuring the advantages of the heterointerfaces. Furthermore, most reported materials are limited to oxide-based heterostructures (ZnO/CuO [28], TiO_2_/Cu_2_O [50], and MnO_2_/ZnO [51]), whereas the low Li-ion conductivity of Li_2_O formed during the pre-lithiation process is omitted.

Inspired by the above discussion, we successfully developed a novel tin phosphide (SnP_0.94_)/cobalt phosphide (CoP) heterostructure (SCP) as the modulation layer for stable LMAs. The SCP was engineered meticulously to display a well-defined heterointerface, an extensive exposure of Sn/Co-P bonds, and a porous structure. This unique composition and heterostructure of the SCP have the following advantages: (1) The enhanced porosity of the SCP mitigates the local current density, while the increased exposure of Sn/Co-P relative to Sn/Co–O amplifies the advantageous heterointerface effect. (2) The heterointerface between CoP (*p*-type semiconductor) and SnP_0.94_ (*n*-type semiconductor) forms a *p*-*n*-type heterojunction. This results in the generation of FIDR and BIEF. FIDR provides richer Li adsorption sites and higher Li adsorption energy. BIEF facilitates electron redistribution and Li-ion migration. (3) In terms of chemical composition, both CoP and SnP_0.94_ have significantly higher Li binding energies than metallic Li. Thus, the synergistic effect between the lithiophilic CoP/SnP_0.94_ and their heterostructure resulted in significantly enhanced Li deposition kinetics and a more uniform Li deposition behavior. Furthermore, during the repetitive Li plating/stripping process, lithiophilic Li_*x*_Sn, electronically conductive Co, and Li-ion conductive Li_3_P formed by the spontaneous reaction between the SCP and metallic Li provided an MEIC layer. This ensured a stable Li deposition behavior during long-term cycling. With the aforementioned advantages, SCP@Li accomplishes dendrite-free Li deposition with an ultra-low nucleation overpotential (~2.2 mV) and the suppressed volume expansion even under a high areal capacity (5 mAh cm^−2^). More remarkably, the symmetric cell with SCP@Li could be cycled stably 750 times even at a high current density of 5 mA cm^−2^. The SCP@Li anode exhibited superior capacity and cycling stability in LiFePO_4_ (LFP) and LiNi_0.8_Co_0.1_Mn_0.1_O_2_ (NCM811) full cells. The design strategy of heterostructured SCP as modulation layer on LMAs significantly contributes the enhancement of cell performance.

## Experimental Section

### Preparation of Heterostructured SnP_0.94_/CoP (SCP)

First, SnCo(OH)_6_, a precursor of SCP, was synthesized using a co-precipitation method. Two millimoles each of Co(NO_3_)_2_·6H_2_O (+ 98%, Sigma-Aldrich) and C_6_H_5_Na_3_O_7_ (99%, Alfa Aesar) were stirred in 70 mL of deionized water (DW) for 30 min (solution A). Ten milliliters of 0.2 M SnCl_4_·5H_2_O (98%, Sigma-Aldrich) solution was added dropwise to solution A under vigorous stirring for 30 min (solution B). To adjust the pH to approximately 10, 10 mL of 2 M NaOH (98%. Samchun Chemicals) was added gradually to solution B using a burette. After vigorously stirring the solution for 1 h (color variation: purple → pink), the pink precipitates were obtained by centrifuging and rinsing with DW and ethanol solution, and dried at 70 °C for 24 h in a convection oven.

Second, the collected SnCo(OH)_6_ products were mixed with NaH_2_PO_2_ using mortar and pestle for 5 min (molar ratio of SnCo(OH)_6_:NaH_2_PO_2_ = 1:10) and heat-treated at 280 °C for 10 min at 10 °C min^−1^ with flowing Ar gas (20 sccm). After natural cooling, the black products were ground and washed using a 0.05 M HCl (36%, Samchun Chemicals) solution and DW. Finally, SCP was obtained after drying at 70 °C for 24 h in a convection oven.

### Preparation of SCP Gas-phase Phosphidation

The SnCo(OH)_6_ precursor and NaH_2_PO_2_ (molar ratio of SnCo(OH)_6_:NaH_2_PO_2_ = 1:10) were placed at the center and in the upstream zone of a quartz tube, respectively, and heat-treated to 300 °C for 3 h with flowing Ar gas (20 sccm). The as-synthesized SCP gas-phase products were collected directly without additional washing.

### Preparation of CoP

First, Co(OH)_2_, a precursor of CoP, was synthesized via precipitation and hydrothermal methods. Ten millimoles of Co(NO_3_)_2_·6H_2_O was dissolved in 20 mL DW. Then, 10 mL of 2 M NaOH solution was added (formation of precipitate). The solution was transferred to 60 mL Teflon-lined autoclave and heated for 2 h at 120 °C. The synthesized products were washed with DW and dried at 70 °C for 24 h in a convection oven.

Second, similar to the SCP synthesis, Co(OH)_2_ was mixed with NaH_2_PO_2_ using a mortar and pestle for 5 min (molar ratio of Co(OH)_2_:NaH_2_PO_2_ = 1:10) and heat-treated under conditions similar to those for SCP. After natural cooling, the black products were ground and washed using a 0.05 M HCl solution and DW. Finally, CoP was obtained after drying at 70 °C for 24 h in a convection oven.

### Preparation of SnP_0.94_

First, SnO_2_, a precursor of SnP_0.94_, was synthesized using precipitation and hydrothermal methods. Next, 10 mmol of SnCl_4_·5H_2_O was dissolved in 20 mL of DW, and 10 mL of 2 M NaOH solution was added (formation of precipitate). The solution was transferred to a 60 mL Teflon-lined autoclave and heated for 12 h at 160 °C. The synthesized products were washed with DW and dried at 70 °C for 24 h in a convection oven.

Second, similar to the SCP synthesis, SnO_2_ was mixed with NaH_2_PO_2_ using a mortar and pestle for 5 min (molar ratio of SnO_2_:NaH_2_PO_2_ = 1:100) and heat-treated under conditions similar to those for SCP. After natural cooling, the black products were ground and washed using a 0.05 M HCl solution and DW. Finally, SnP_0.94_ was obtained after drying at 70 °C for 24 h in convection oven.

### Materials Characterization

Field-emission scanning electron microscope (FESEM, Hitachi, SU-70) was used to analyze the morphology of SnCo(OH)_6_, SCP, CoP, and SnP_0.94_. The crystal structure was analyzed by X-ray diffraction (XRD, Rigaku, Miniflex 600 diffractometer) with Cu Kα radiation at a scanning rate of 2° min^−1^. The Brunauer–Emmett–Teller (BET) surface areas and Barrett-Joyner-Halenda (BJH) pore size distributions were obtained using an N_2_ adsorption–desorption process (MicrotracBEL Corporation, BELSORP-mas instrument) to compare the surface areas and pore distributions of SnCo(OH)_6_ and SCP. The microstructure and elemental distribution of the SCP were observed using field-emission transmission electron microscope (FE-TEM, JEOL, JEM-F200) with energy-dispersive X-ray spectroscopy (EDS) detector. X-ray photoelectron spectroscopy (XPS, Thermofisher, Nexsa) with Al Kα radiation was applied to verify the chemical compositions and elemental valences. To determine the work functions of materials, UV photoelectron spectroscopy (UPS, Thermofisher, theta probe base system) measurements were performed with a helium discharge lamp (ℎν = 21.2 eV). The cross-sectional SEM and EDS images were captured after argon milling (accelerating voltage of 2 kV) using a cross-sectional ion polisher (JEOL, IB-19520CCP).

### Electrochemical Measurements

Firstly, the modified Li anodes with modulation layers were fabricated through drop casting method. The synthesized SnP_0.94_, CoP, and SCP powders (10 mg) were stirred vigorously in 1 mL of toluene (99.8%, Sigma-Aldrich) for 3 h, and then 60 uL of the solution was dropped onto Li. After drying under vacuum at room temperature for 6 h, the modified Li anodes were obtained. All electrochemical characterization were conducted using CR2032-type coin cell, ether-based electrolyte (1 M lithium bis(trifluoromethanesulfonyl)imide (LiTFSI, 99.95%, Sigma-Aldrich) with 2 wt% LiNO_3_ (99.99%, Sigma-Aldrich) in dioxolane (DOL, 99.8%, Sigma-Aldrich)/dimethoxyethane (DME, 99.5%, Sigma-Aldrich) at a volume ratio of 1:1), and a polypropylene separator (Celgard 2400). Galvanostatic and cyclic voltammetry (CV) tests were measured using an automatic battery cycler (WBCS 3000, WonATech Co.). Electrochemical impedance spectroscopy (EIS) profiles were recorded in the frequency range of 100 kHz to 0.01 Hz with an alternating voltage amplitude of 10 mV using IVIUMnSTAT electrochemical analyzer (IVIUM Technologies). For CE tests of asymmetric cells (Li//Cu), the charge cutoff voltage was set to 0.5 V *vs.* Li/Li^+^. For full cell tests, LiFePO_4_ (LFP, 1 C = 170 mAh g^−1^) and LiNi_0.8_Co_0.1_Mn_0.1_ (NCM811, 1 C = 200 mAh g^−1^) as cathode materials was coated on an Al current collector with a homogeneously mixed slurry composed of LFP or NCM811:Super P carbon black (Alfa Aesar):polyvinylidene fluoride (PVDF, Kynar 2801) in a weight ratio of 8:1:1. The coated electrodes was dried at 70 °C for more than 8 h under vacuum. All full cells were activated for 1 cycle at 0.1 C. The mass loading of active materials (LFP and NCM811) was ~3.0 mg cm^−2^. The galvanostatic charge/discharge tests of NCM811//Li full cells were measured in the potential window of 3.0–4.3 V (vs. Li/Li^+^). For LFP//Li full cells, the potential window was set from 2.5 to 4.2 V (vs. Li/Li^+^).

### Measurement of Li^+^ Diffusion Coefficient

To measure the Li^+^ diffusion coefficient (${\text{D}}_{{\text{Li}}^{+}}$), CV tests were conducted on the full cells at various scan rates (0.1, 0.2, 0.4, 0.6, 0.8, and 1.0 mV s^−1^). ${\text{D}}_{{\text{Li}}^{+}}$ was calculated by the Randles–Sevcik equation (Eq. 1) using the obtained CV curves that exhibited a linear relationship between the peak current (*I*_*peak*_) and square root of the CV scan rate (*v*^*0.5*^).

1 $$I_{{{\text{peak}}}} = 2.69 \times 10^{5} n^{1.5} {\text{AD}}_{{{\text{Li}}^{ + } }}^{0.5} {\text{C}}_{{{\text{Li}}^{ + } }} v^{0.5}$$

where *n* is the number of electrons transferred, *A* is the surface area of the electrode, ${\text{C}}_{{\text{Li}}^{+}}$ is the Li-ion concentration in the electrolyte, and v is the voltage scan rate.

### Measurement of Ionic Conductivity

The ionic conductivity (*σ*) of the SCP modulation layer was measured by EIS using stainless steel (SS)|SCP coated glass fiber (GF/F, Whatman)|SS. The reason for using the SCP layer coated on GF via the vacuum filtration method was to measure the ionic conductivity of the SCP alone, without the influence of any binder. To compare the ionic conductivities of the SCP layers, SS|GF|SS was used as a blank cell. *σ* is calculated as follows Eq. 2.

2 $$\sigma = \frac{l}{{R_{{\text{b}}} S}}$$

where* l* is the thickness of the separator, *R*_b_ is the bulk resistance, and *S* is the contact area of the SS.

### Measurement of Activation Energy

To determine the activation energy of the SCP modulation layer, the ionic conductivity was measured at different temperatures. The activation energy (E_a_) was calculated from a linear fit to the Arrhenius equation (Eq. 3):

3 $$\sigma = \sigma_{0} \times e^{{\left( { - E_{{\text{a}}} /RT} \right)}}$$

where *R* is the gas constant and *T* is the absolute temperature.

### Finite Element Simulation

A finite element simulation was conducted using 3D modeling in COMSOL Multiphysics 6.3 to understand the electrodeposition process. Specifically, a tertiary current distribution module was used to investigate the current density ($${i}_{l})$$ distribution on the LMA surface based on the Nernst equations (Eqs. 4 and 5):

4 $$J_{{{\text{Li}}}} = - D_{{{\text{Li}}}} \nabla c_{{{\text{Li}}}} - z_{{{\text{Li}}}} u_{{{\text{Li}}}} Fc_{{{\text{Li}}}} \nabla \emptyset_{l}$$

5 $$i_{l} = F\Sigma_{{{\text{Li}}}} z_{{{\text{Li}}}} J_{{{\text{Li}}}}$$

where $D_{{{\text{Li}}}}$ $(0.5 \times 10^{ - 9} \,{\text{m}}^{2} \,{\text{s}}^{ - 1} )$, $c_{{{\text{Li}}}}$ (1.0 M), $z_{{{\text{Li}}}}$ (1.0), and $u_{{{\text{Li}}}}$ represent the diffusion coefficient, concentration, charge number, and electric mobility of the Li ions, respectively. $F$ and ${\varnothing }_{l}$ denote the Faraday constant (96,485 C mol^−1^) and the electrolyte potential, respectively.

In the 3D modeling, the lithium metal anode was set to a size of 5 × 4.5 µm^2^. Meanwhile, the diameters of the initial crystal nuclei were set to 0.55 and 0.15 µm for BLi and SCP@Li, respectively. The potential difference between the top and bottom (defined as the cathode and anode, respectively) was set to 0.02 V.

The electrochemical reaction for Li deposition on the electrode surface follows the Butler–Volmer equation (Eq. 6) shown below:

6 $$i_{{\text{Li }}} = i_{0} \left[ {\exp \left( {\frac{{\alpha_{{\text{a}}} F\eta }}{{{\text{RT}}}}} \right) - \exp \left( { - \frac{{\alpha_{{\text{c}}} F\eta }}{{{\text{RT}}}}} \right)} \right]$$

where ${i}_{0}$ is the exchange current density (${i}_{0}$= 3 mA cm^−2^), ${\alpha }_{a}$ is the anodic charge transfer coefficient (${\alpha }_{a}$= 1.5), ${\alpha }_{c}$ is the cathodic charge transfer coefficient (${\alpha }_{c}$= 0.5), and $\eta$ is the overpotential.

### Density Functional Theory (DFT) Computational Methods

First-principles calculations were carried out using the projector augmented wave method and the Perdew–Burke–Ernzerhof (PBE) generalized gradient approximation, as implemented in the Vienna Ab Initio Simulation Package (VASP). A plane wave basis set with a cutoff energy of 520 eV was employed for the calculations, and a gamma-centered 2 × 2 × 1 grid was utilized for the k-point sampling. The electronic self-consistency and ionic relaxation loops were converged with criteria of 10^−5^ eV and 0.03 eV Å^−1^, respectively. All calculations were spin-polarized, and van der Waals (vdW) interactions were accounted for using the DFT-D3 correction method. The calculations utilized the experimentally observed CoP(011) and SnP_0.94_(011) slabs. The adsorption energy of Li, *E*_ads_Li_, was calculated using the formula ${E}_{\text{ads}\_\text{Li}}={E}_{\text{all}}-{E}_{\text{substrate}}-{E}_{\text{Li}}$, where ${E}_{\text{all}}$, ${E}_{\text{substrate}}$, and ${E}_{\text{Li}}$ represent the total energies of all system, substrate, and Li atom, respectively. The charge density difference for the formation of the CoP(011)/SnP_0.94_(011) heterostructure, $\Delta {\rho }_{\text{heterostructure}}$, was calculated using the formula $\Delta {\rho }_{\text{heterostructure}}={\rho }_{\text{all}}-{\rho }_{\text{CoP}(011)}-{\rho }_{\text{SnP}0.94(011)}$, where ${\rho }_{\text{all}}$, ${\rho }_{\text{CoP}(011)}$ and ${\rho }_{\text{SnP}0.94(011)}$ represent the charge densities of all system, the CoP(011) component, and SnP_0.94_(011) component, respectively. Bader charge analysis was conducted using the Henkelman group’s Bader charge analysis code to examine charge transport in the CoP(011)/SnP_0.94_ (011) heterostructure.

## Results and Discussion

### Fabrication of Heterostructured Tin Phosphide (SnP_0.94_) and Cobalt Phosphide (CoP)

**Fig. 1:**
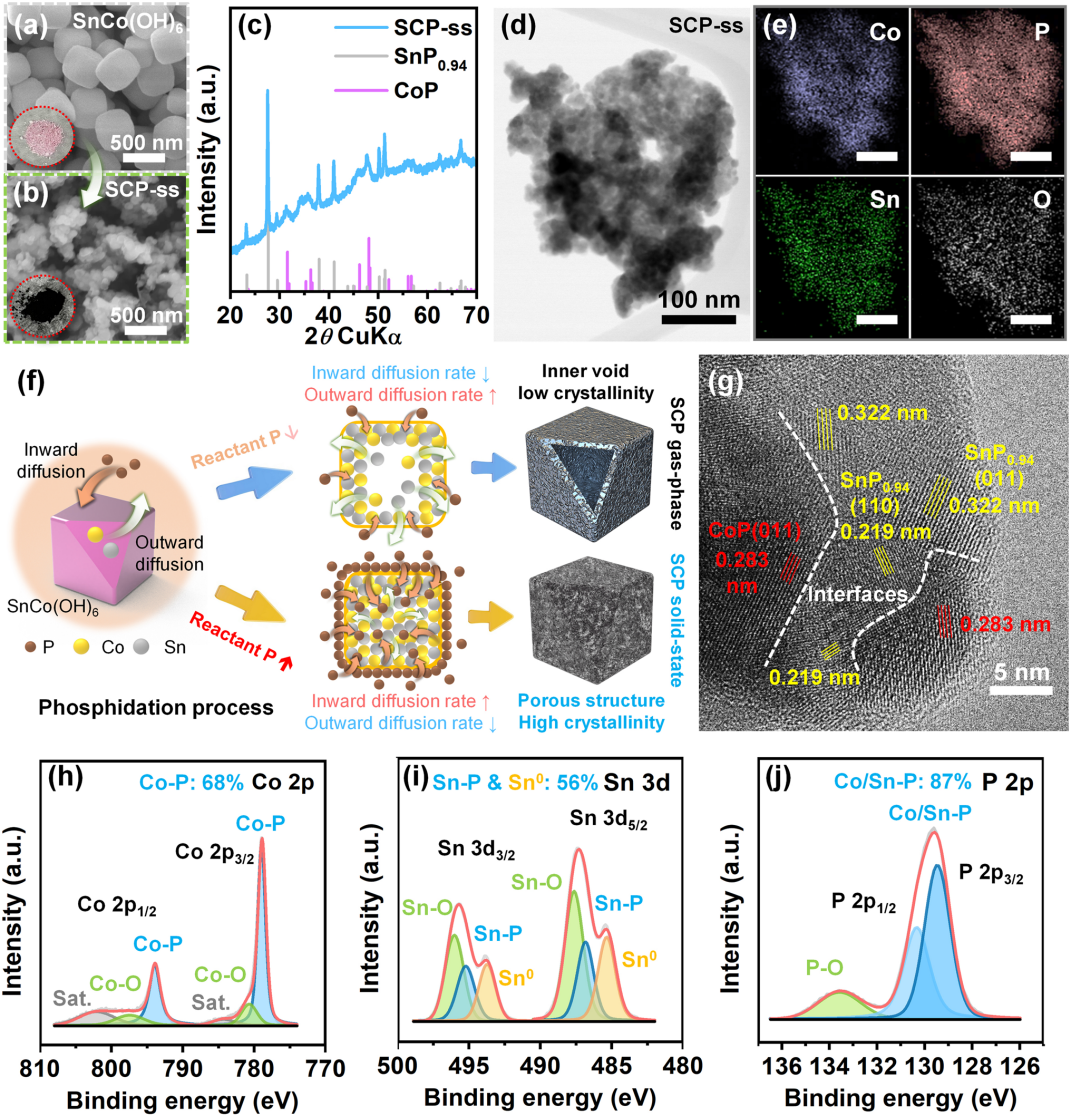
Synthesis and characteristics of SCP. SEM images of **a** SnCo(OH)_6_ and **b** SCP-ss (inset: digital images of SnCo(OH)_6_ and SCP-ss powders). **c** XRD pattern of SCP-ss. **d** TEM image and **e** corresponding EDS elemental mapping images (scale bar: 100 nm) of SCP-ss. **f** Schematic illustration of the synthesis process of SCP according to the Kirkendall effect. **g** High-resolution TEM image of SCP-ss. XPS spectra of **h** Co 2*p*, **i** Sn 3*d*, and **j** P 2*p* of SCP-ss

The unique SCP with SnP_0.94_/CoP heterojunctions was fabricated using a two-step fabrication process. First, SnCo(OH)_6_ nanocubes, as precursors of SCP, were synthesized via a co-precipitation method (the procedures are detailed in the Experimental Section). After solid-state phosphidation (not gas-phase phosphidation with PH_3_) of SnCo(OH)_6_ at low temperature (280 °C) for a short time (10 min) by mixing with sodium hypophosphite, a heterostructured porous SCP was fabricated successfully with multitudinous advantages for LMAs. Figures 1a and S1a show a scanning electron microscope (SEM) image and X-ray diffraction (XRD) pattern of the SnCo(OH)_6_ intermediate products during the SCP fabrication process, respectively. The pink SnCo(OH)_6_ exhibited a well-defined nanocube morphology (average particle size =  ~300 nm) and typical XRD pattern indexed to the perovskite hydroxide phase (PDF No. 26-1401). For the phosphidation of SnCo(OH)_6_, we introduced a solid-state phosphidation process (Eq. 7) [52] rather than the conventional gas-phase phosphidation process (Eqs. 8–11) [53] to simultaneously form a porous and clear heterogeneous phase of SCP.

7 $${\text{SnCo}}({\text{OH}})_{6} ({\text{s}}) + x{\text{NaH}}_{2} {\text{PO}}_{2} ({\text{s}}) \to {\text{SnP}}_{y} ({\text{s}}) + {\text{CoP}}_{z} ({\text{s}}) + x{\text{NaOH}}({\text{s}}) + \gamma {\text{H}}_{2} {\text{O}}({\text{l}})$$

8 $$3{\text{NaH}}_{2} {\text{PO}}_{2} ({\text{s}}) \to {\text{PH}}_{3} ({\text{g}}) + {\text{NaH}}_{2} {\text{PO}}_{3} ({\text{s}}) + {\text{Na}}_{2} {\text{HPO}}_{3} ({\text{s}})$$

9 $$4{\text{NaH}}_{2} {\text{PO}}_{3} ({\text{s}}) \to {\text{PH}}_{3} ({\text{g}}) + 2{\text{NaH}}_{2} {\text{PO}}_{4} ({\text{s}}) + {\text{Na}}_{2} {\text{HPO}}_{4} ({\text{s}})$$

10 $$4{\text{Na}}_{2} {\text{HPO}}_{3} ({\text{s}}) \to {\text{PH}}_{3} ({\text{g}}) + {\text{Na}}_{2} {\text{HPO}}_{4} ({\text{s}}) + 2{\text{Na}}_{3} {\text{PO}}_{4} ({\text{s}})$$

11 $${\text{SnCo}}({\text{OH}})_{6} ({\text{s}}) + x{\text{PH}}_{3} ({\text{g}}) \to {\text{SnP}}_{y} ({\text{s}}) + {\text{CoP}}_{z} ({\text{s}}) + \gamma {\text{H}}_{2} {\text{O}}(l)$$

For SCP synthesized via the gas-phase phosphidation process (denoted as SCP-gp), although the overall outer shape of SnCo(OH)_6_ nanocubes was maintained, the nanoparticles contracted to a smaller size (~200 nm) with numerous nanosized bumps covering the surface of SCP-gp (Fig. S2a). The XRD pattern of SCP-gp reveals an amorphous structure without observable heterophase crystallinity (Fig. S2b). Notwithstanding the more intense thermal treatment conditions (300 °C and 3 h) of the gas-phase phosphidation process than those of the solid-state phosphidation process (280 °C and 10 min), the energy-dispersive X-ray spectroscopy (EDS) spectrum of SCP-gp exhibits a significantly low phosphorus-to-oxygen atomic ratio (P: 6.9 at%, O: 47.8 at%) (Fig. S3a). The amorphous structure and high oxygen content of SCP-gp imply a limited reactant P (sourced from PH_3_ gas) concentration and low reactivity. Consequently, this does not satisfy the requirements for a distinct heterostructure and a high P/O ratio in the SCP. In contrast, the SEM image of the SCP synthesized via the solid-state phosphidation process (denoted as SCP-ss) displays a significantly reduced particle size (~150 nm) and porous structure (Figs. 1b and S4). Additionally, it exhibits a well-defined heterophase crystalline structure of SnP_0.94_ (PDF No. 01-080-1201) and CoP (PDF No. 62-4588) compared with the amorphous SCP-gp (Fig. 1c). The enhanced porosity of SCP-ss was verified further by Brunauer–Emmett–Teller (BET) and Barrett-Joyner-Halenda (BJH) analyses (Fig. S5). SCP-ss demonstrates specific surface area (12.74 m^2^ g^−1^) and pore volume (0.1033 cm^3^ g^−1^) higher than those of SnCo(OH)_6_ (7.64 m^2^ g^−1^ and 0.0467 cm^3^ g^−1^) and SCP-gp (5.21 m^2^ g^−1^ and 0.0575 cm^3^ g^−1^). This is favorable for inducing evenly Li-ion deposition and decreasing the local current density [4, 54]. Notably, in the inductively coupled plasma-optical emission spectrometer (ICP-OES) measurement and EDS spectrum (Figs. S6 and S3b), SCP-ss maintains the atomic ratio of Sn and Co at 1:1 but differs significantly from SCP-gp in that the proportion of P element (43.3 at%) is much higher than that of O element (14.1 at%). Therefore, the distinct heterophase crystalline structure, enhanced porosity, and abundant P content of SCP-ss make it a more potential candidate as a heterostructured material for facilitating stable Li behavior [42, 47]. To analyze the deeper microstructure and elemental distribution of SCP, transmission electron microscope (TEM) with EDS was used. As shown in Figs. 1d, e, and S7, SCP-ss was composed of a multitude of nanobubbles with a mean diameter of around 20 nm, and Co, Sn, P, and O were distributed uniformly without phase separation. The nanobubble structure observed after phosphidation originates from the nanoscale Kirkendall effect (Fig. 1f). During phosphidation process, Sn and Co ions typically exhibit an outward diffusion tendency to react with P to form SnP_x_ and CoP_x_. Meanwhile, the diffusion of P ions is primarily inward, and driven by chemical potential and concentration gradients [55, 56]. As shown in Fig. S8, at low concentrations of reactant P (SCP-gp), the phosphidized product formed hollow inner voids owing to the diffusion rate of outward species (Sn and Co) being higher than that of the inward species (P). This is attributed to an insufficient concentration gradient of reactant P, which limits its rapid inward diffusion. Otherwise, at high concentrations of reactant P (SCP-ss), the significantly higher diffusion rate of the inward species than that of the outward species resulted in the formation of porous structures combined with numerous nanopores and nanobubbles within SCP-ss via the Kirkendall effect in localized areas (Figs. 1d and S7). Furthermore, the high-resolution TEM image of SCP (Fig. 1g) shows distinct interfaces between the lattice fringes of the SnP_0.94_ ((110) and (011) crystal planes, corresponding to interplanar distances of 0.219 and 0.322 nm, respectively) and lattice fringe CoP ((011) crystal plane, corresponding to an interplanar distance of 0.283 nm), indicating the presence of SnP_0.94_/CoP heterointerface. The fast Fourier transform (FFT) pattern (Fig. S9) also demonstrates distinguishable lattices corresponding to the SnP_0.94_(011), CoP(011), SnP_0.94_(110), and CoP(211) planes, from the innermost to the outermost rings, respectively.

X-ray photoelectron spectroscopy (XPS) was used to analyze the elemental composition and valence states of SCP-ss. The Co 2*p* spectrum (Fig. 1h) exhibited three distinct spin-orbital split peaks (Co 2*p*_1/2_ and Co 2*p*_3/2_). The dominant component is at 793.8 and 778.9 eV corresponding to the Co-P bond in the Co^3+^ oxidation state [57, 58]. It accounts for 68% of the spectral area. Two additional sets of peaks at 798.5/780.6 eV and 803.3/784.2 eV correspond to the Co–O bond (Co^2+^) and satellite peaks, respectively [59, 60], accounting for ~17% and ~15% of the spectral area. The Sn 3*d* spectrum (Fig. 1i) also shows three sets of doublet peaks (Sn 3*d*_3/2_ and Sn 3*d*_5/2_) at 495.6/487.2 eV, 494.8/486.4 eV, and 493.3/484.9 eV, corresponding to Sn–O (Sn^4+^), Sn-P (Sn^2+^), and Sn metal (Sn^0^), respectively [61, 62]. The presence of the Sn^0^ peak is attributed to the metallic β-Sn-like bonding in the SnP_0.94_ crystal structure [63, 64]. Thus, similar to the Co 2*p* spectrum, the fraction of valence states associated with SnP_0.94_ (Sn^2+^ and Sn^0^) is ~56%. It is more abundant than the Sn-O bonds. The P 2*p* spectrum (Fig. 1j) is deconvoluted into three peaks at 133.5, 130.3, and 129.4 eV, corresponding to P-O, Co/Sn-P (P 2*p*_1/2_), and Co/Sn-P (P 2*p*_3/2_) bonds, respectively [42, 58]. Although superficial oxidation results in the formation of P-O (~13% in the P 2*p* spectral area), Co-O, and Sn-O bonds, the degree of superficial oxidation in the SCP-ss is significantly lower than that in SCP-gp (Fig. S10) and previously reported transition metal phosphides synthesized via the gas-phase phosphidation process [42, 45, 65]. This indicates a higher density of exposed Sn/Co–P bonds and their interfaces in SCP-ss. Based on a comparative study between SCP-ss and SCP-gp, we designated SCP-ss simply as SCP hereafter.

### Understanding the Band Structure and Its Impact in SCP

**Fig. 2:**
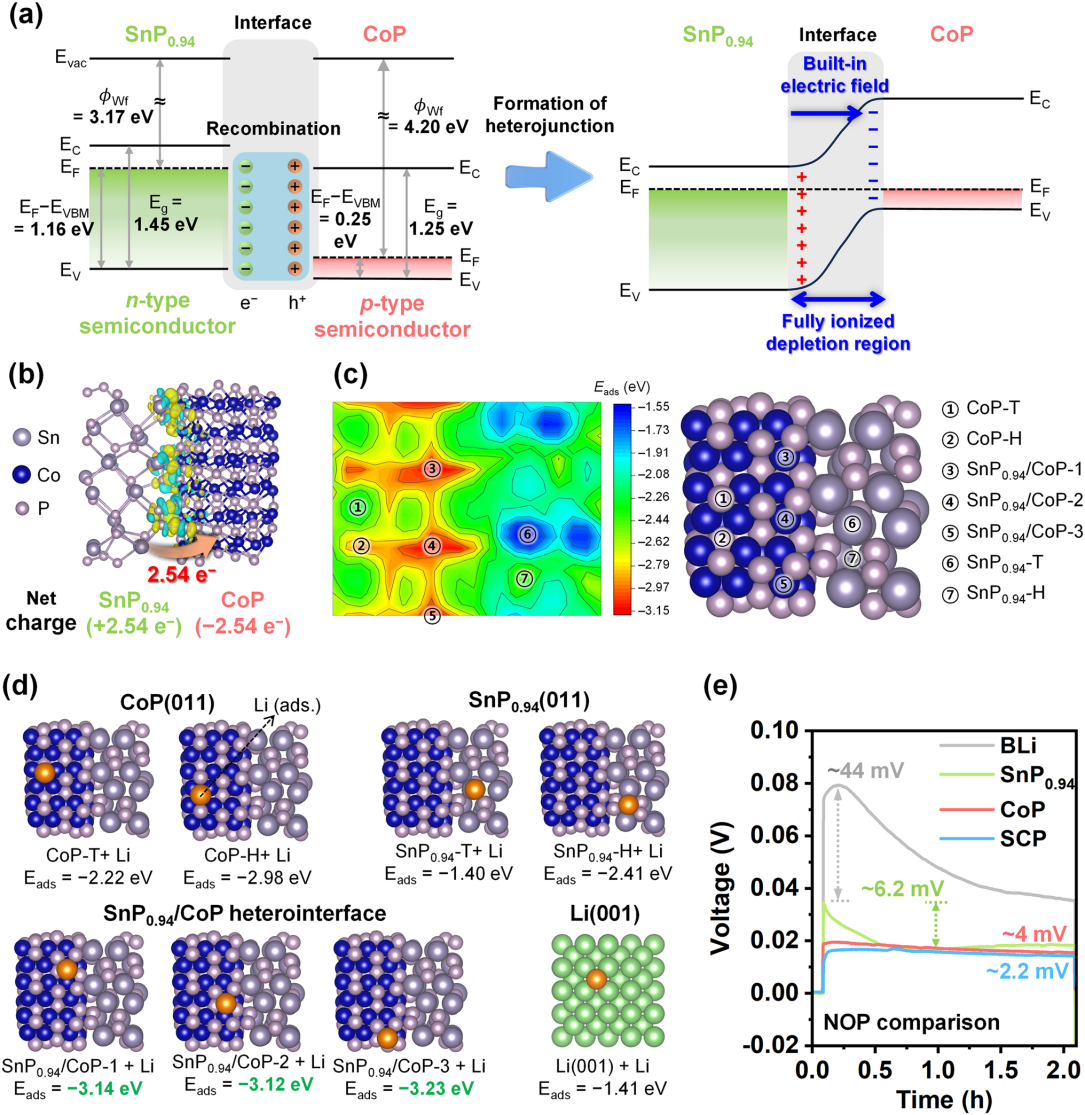
Band structure and its impact evaluation for SCP. **a** Schematic diagram of energy levels before and after contact between SnP_0.94_ and CoP. **b** Differential charge density of SnP_0.94_/CoP heterointerface (yellow area: electron accumulation, cyan area: electron depletion). **c** Contour plot of adsorption energy of Li on SnP_0.94_/CoP heterostructure. **d** E_ads_ on Li(001), CoP(011), SnP_0.94_(011), and CoP(011)/SnP_0.94_(011) heterointerface. **e** Voltage–time profiles of BLi, SnP_0.94_@Li, CoP@Li, and SCP@Li at a current density of 0.5 mA cm^−2^

The distinctive characteristics of heterostructures are not realized only through the formation of heterojunctions between different materials. Typically, the unique merits of heterostructures are closely linked to specific microstructures with distinct band structures of the heterointerfaces. Therefore, we analyzed the band structures of SnP_0.94_ and CoP to evaluate whether SCP can provide favorable heterointerfaces. SnP_0.94_ and CoP were prepared through a solid-state phosphidation process analogous to that for SCP (Fig. S11). To thoroughly investigate the band structure of SnP_0.94_ and CoP, the work function ($\phi_{{{\text{Wf}}}}$), valence band maximum (*E*_VBM_), and band gap (*E*_g_) energies of each material were obtained by ultraviolet photoelectron spectroscopy (UPS) spectra, XPS valence band spectra, and UV–visible absorption spectra, respectively (Fig. S12). The energy positions of $\phi_{{{\text{Wf}}}}$, E_VBM_, and E_g_ for SnP_0.94_ and CoP are summarized in Table S1. Based on the calculated energy levels of SnP_0.94_ and CoP, the energy diagrams before and after the contact between SnP_0.94_ and CoP are illustrated in Fig. 2a. The band diagram shows that SnP_0.94_ is an *n*-type semiconductor with E_F_ higher than that of CoP. Meanwhile, CoP is a *p*-type semiconductor with E_F_ lower than that of SnP_0.94_. Therefore, after SnP_0.94_ and CoP come into contact, the energy bands at the heterointerfaces spontaneously align until the E_F_ of the two materials attains equilibrium, as the electrons (the majority carriers of the *n*-type semiconductor) of SnP_0.94_ and the holes (the majority carriers of *p*-type semiconductor) of CoP recombine at the heterointerfaces [66]. As a result, the partially intrinsic electroneutral region near the heterointerfaces is ionized by formatting the FIDR and generate to charge separation. This, in turn, yields region of localized positive and negative charge [67]. These charged regions function as active sites for electrochemical reactions and effectively adsorb oppositely charged species. In addition, the charge separation region in the FIDR leads to BIEF, thereby facilitating charge redistribution by the additional electric field force. This effectively distributes the charge density and alleviates the intense local current density. The charge redistribution and beneficial effects at the SCP heterointerfaces were further confirmed by density functional theory (DFT) calculations. We set SnP_0.94_(011) and CoP(011) as representative crystal planes based on the exposed crystal planes observed in the TEM image of the SCP (Fig. 1g). The differential charge density simulation (Fig. 2b) at SnP_0.94_/CoP heterointerface demonstrated that a net electron of 2.54*e* according to the Bader analysis transfers from SnP_0.94_ to CoP [68]. This is consistent with the calculated band structure model (Fig. 2a). Therein, the n-type semiconductor SnP_0.94_ (displaying E_F_ higher than that of CoP) functions an electron donor. The interactions between Li atoms and the SnP_0.94_, CoP, and SnP_0.94_/CoP heterointerface were also calculated to evaluate their lithiophilicity. The adsorption sites of Li atoms (E_ads_) on SnP_0.94_, CoP, and SnP_0.94_/CoP heterointerface were selected after an initial screening of adsorption trends on the surface (Fig. 2c). As shown in Fig. 2d, E_ads_ of SnP_0.94_ (SnP_0.94_-H: −2.41 eV), CoP (CoP-T/H: −2.22/−2.98 eV), and SnP_0.94_/CoP heterointerface (SnP_0.94_/CoP-1/2/3: −3.14/−3.12/−3.23 eV) are significantly higher than the Li cohesive energy (−1.41 eV), indicating that SnP_0.94_, CoP, and their heterointerface can provide favorable nucleation sites to lower Li nucleation barrier. Interestingly, among them, the highest E_ads_ of SnP_0.94_/CoP heterointerface suggests that the well-designed heterointerface can supply more Li adsorption sites and enhance the Li nucleation kinetics more than SnP_0.94_ or CoP [49]. Profiting from the abundant Li adsorption sites and superior Li nucleation kinetics of SnP_0.94_/CoP heterointerface based on DFT calculation, we evaluated the nucleation overpotential (NOP) of the Li anodes applied with SCP as modulation layer (SCP@Li) at a current density of 0.5 mA cm^−2^ (Fig. 2e). SCP@Li exhibits an exceptionally reduced NOP (~2.2 mV) compared with those of bare Li (BLi, ~44 mV), SnP_0.94_@Li (~16.2 mV), and CoP@Li (~4 mV). It has a trend similar to that of the DFT calculations of E_ads_. This implies that SnP_0.94_, CoP and SCP can all effectively lower the Li nucleation barrier more than BLi, but SCP with heterointerface can further promote the phase transition of Li ions on the SCP surface. Furthermore, as shown in Fig. S13, at the Li growth step corresponding to the steady-state voltage, the lowest overpotential of SCP@Li also indicates that the SCP markedly helps reduce the voltage polarization and improve the Li deposition kinetics during the Li deposition process.

### Revealing the Li Deposition Mechanism on SCP@Li

**Fig. 3:**
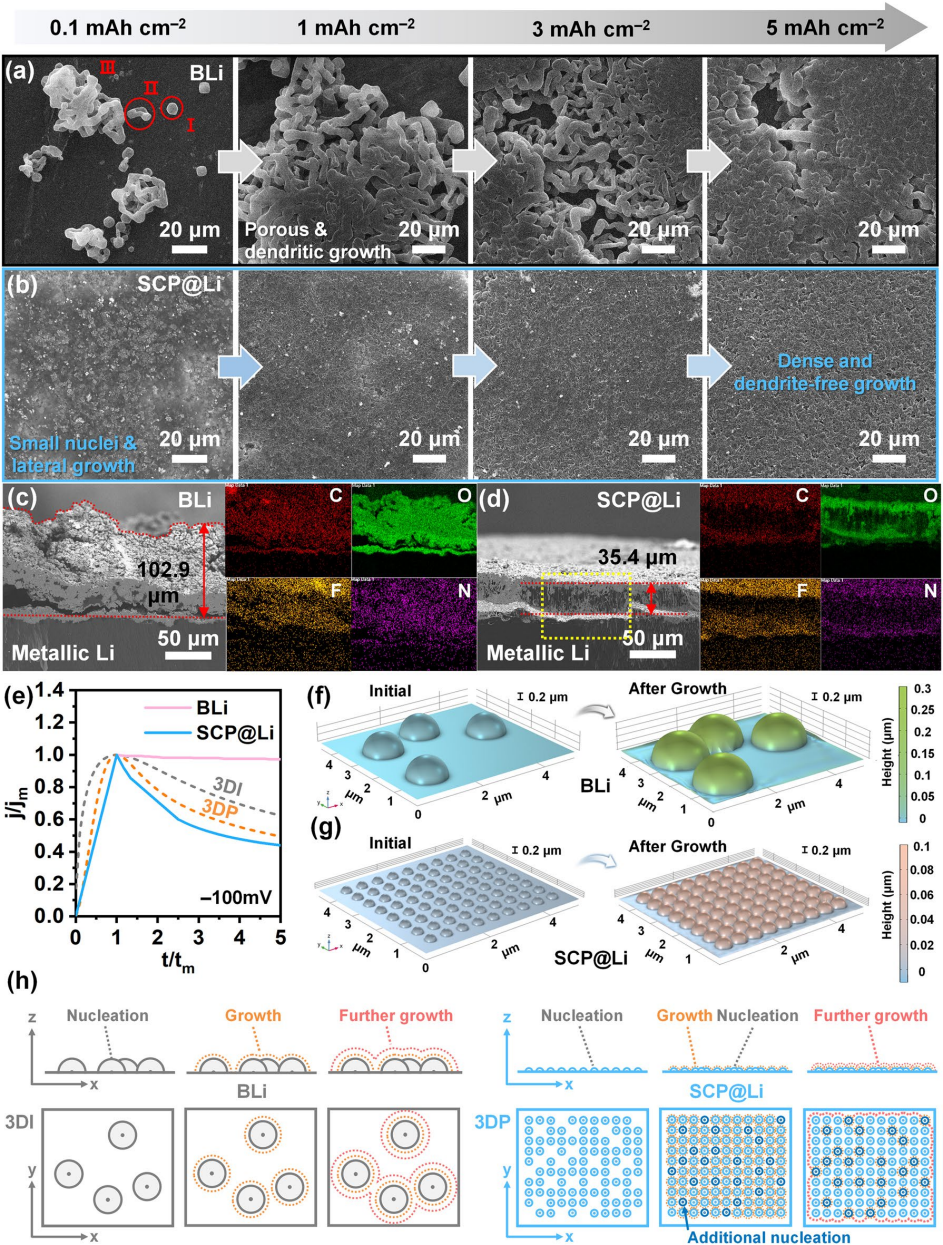
Li deposition behavior of SCP@Li. Ex-situ SEM images of **a** BLi and **b** SCP@Li after Li deposition at a fixed current density of 1 mA cm^−2^ with areal capacities of 0.1, 1, 3, and 5 mAh cm^−2^. Cross-sectional BSE and EDS images of **c** BLi and **d** SCP@Li after Li deposition of 5 mAh cm^−2^. **e** Dimensionless plot of experimental current transients of Li metal deposition using BLi and SCP@Li. Simulated initial nuclei models and final Li deposition models of **f** BLi and **g** SCP@Li after 100 s of Li deposition. **h** Schematic illustration of the Li nucleation and growth mechanism of BLi and SCP@Li

To scrutinize how the SCP modulation layer affects the initial nucleation and further growth of Li, the Li deposition behavior as a function of the stepwise deposition capacity was analyzed by ex-situ SEM. Surface and cross-sectional SEM images of the pristine SCP@Li show that the SCP layer is formed with a thickness of ~ 11 μm without cracks on the Li foil through optimized coating conditions (Fig. S14). When Li of 0.05 mAh cm^−2^ were deposited on BLi and SCP@Li at a current density of 1 mA cm^−2^, the initial Li nucleation behaviors of BLi and SCP@Li showed significant differences (Fig. S15). The huge hexagonal initial Li nuclei (~4.7 μm) on BLi (Fig. S15a) can be attributed to the preferential growth of metallic Li (body-centered cubic (bcc) crystal structure) along the [111] direction, which minimizes the surface energy by maximizing the exposure of the {110} planes [69]. In contrast, in SCP@Li (Fig. S15b), metallic Li is deposited uniformly on the SCP surface composed of countless SCP nanoparticles (~150 nm), forming small Li nuclei and smooth surface without generating prominent micro-sized Li nuclei. As shown in Figs. 3a, b, and S16, the difference between the initial nucleation steps of BLi and SCP@Li had a significant impact on the subsequent Li growth behavior. After Li deposition of 0.1 mAh cm^−1^, BLi gradually formed dendritic and porous Li deposits in three steps (I: Li nucleation; II: heterogeneous growth around the nuclei; and III: entanglement through continuous growth). The patchy and dendritic Li growth on BLi is mainly attributed to the limited number of Li nucleation sites and low surface Li diffusivity, which favorably deposited Li ions locally at the initial nucleation sites with an increased local current density [5]. This malicious Li deposition behavior of BLi persists up to 5 mAh cm^−2^. The highly porous Li deposits affect the increase in voltage polarization and decrease in cell lifetime because the pores devour a considerable amount of electrolyte (causing electrolyte dry-out and violent side reactions) and accelerate the volume expansion of the LMA during repetitive Li plating/stripping [70]. For SCP@Li, with the altered Li nucleation behavior, lateral Li growth dominates after Li deposition of 0.1 mAh cm^−2^, and is sustained up to 5 mAh cm^−2^. This leads to the formation of dense and dendrite-free Li deposits. In addition, as shown in Fig. S16, the grain size of SCP@Li is observably smaller than that of BLi after Li deposition of 5 mAh cm^−2^, suggesting that the initially small and abundant Li nuclei in SCP@Li significantly influenced the subsequent deposition behavior [2]. The stable Li deposition behavior of SCP@Li is further confirmed even at high current densities (3 and 5 mA cm^−2^). As shown in Fig. S17, BLi still shows non-uniform and island-like Li deposits at low magnification. At high magnification, the surface of Li deposits is composed of clusters of moss-like nanodendrites, unlike under a low current density of 1 mA cm^−2^. The change in Li deposition behavior of BLi under high current densities is evidence for the low surface Li diffusivity and lacking nucleation sites of BLi. This deficiency accelerates the growth of small needle-like dendritic structures and the non-uniformity of Li deposits as the current density increases. In stark contrast, SCP@Li still maintains the dense and dendrite-free Li deposition behavior even under high current densities. The difference in Li deposition kinetics between BLi and SCP@Li becomes more pronounced in the galvanostatic profiles recorded at high current density during Li deposition (Fig. S18). Thus, the stable Li deposition behavior of SCP@Li demonstrates that the SCP modulation layer can provide abundant lithiophilic Li nucleation sites and enhance Li diffusivity. This, in turn, yields a uniform Li-ion/electric field distribution during the Li deposition process. To further investigate the effect of the SCP modulation layer on the Li deposition behavior, cross-sectional backscattered electrons (BSE) and EDS elemental mapping images of BLi and SCP@Li after Li deposition of 5 mAh cm^−2^ were analyzed. The contrast variation in the BSE images (directly related to the difference in atomic number) allows for the distinction between the metallic Li and SEI components. As shown in Fig. 3c, the Li deposits on BLi exhibited a loosely packed chunk morphology and protrusions with a thickness of ~102.9 μm. Also, combined BSE and EDS elemental mapping analyses revealed that the surface of the porous Li deposits was predominantly covered by SEI components, which were formed by the reactions between the electrolyte and metallic Li. The observed results provide compelling evidence of the challenges (electrolyte dry-out, accumulation of SEI components, and volume expansion) associated with the porous Li deposition behavior on BLi. In sharp contrast, the compact Li deposits without protrusions on SCP@Li (thickness: ~35.4 μm) (Fig. 3d) showed an effective suppression of volume expansion during high-capacity Li deposition while maintaining the SCP modulation layer stably (Fig. S19). It is noteworthy that compared with the deposited BLi, significantly few components were obtained from the SEI within the dense Li deposition layer on SCP@Li. This indicates that the Li deposition behavior of SCP@Li can also mitigate the side reactions between the electrolyte and Li deposits. The stable Li deposition behavior of SCP@Li was also verified by constant-current polarization tests using symmetric cells at a current density of 5 mA cm^−2^. In the test, glass fibers (GF/F, Whatman) were used as a separator to analyze the rapid and clear short circuits inside the cell system. As shown in Fig. S20, an abrupt voltage reduction in the symmetric BLi cell (indicating an internal short circuit) was recorded at ~0.24 h. In contrast, SCP@Li sustained Li deposition without observable internal short circuit for ~1.69 h, which was approximately seven times longer than that of the BLi cell. These results strongly support the capability of SCP@Li to guide stable Li deposition even at a high current density.

Given that the electrocrystallization of new phases is closely related to overpotentials, we employed chronoamperometry (CA) technique to record the potentiostatic current transients (Fig. S21). Subsequently, the current transients were normalized to dimensionless transients using the peak current (*j*_*m*_) and its corresponding time (*t*_*m*_) as reference points to gain a deeper insight into the Li nucleation mechanism. A comparison of the dimensionless transients with the Scharifker–Hills model for 3D instantaneous (3DI) and progressive (3DP) nucleation allows for the determination of whether the initial nuclei are generated instantaneously at the beginning or through a progressive process over time. The mathematical equations of the model are as follows (Eqs. 12 and 13) [71]:

12 $$3{\text{DI:}}\quad \left( {\frac{j}{{j_{m} }}} \right)^{2} = 1.9542\left( {\frac{t}{{t_{m} }}} \right)^{{{-}1}} \left\{ {1{-}\exp \left[ {{-}1.2564\left( {\frac{t}{{t_{m} }}} \right)} \right]} \right\}^{2}$$

13 $$3{\text{DP:}}\quad \left( {\frac{j}{{j_{m} }}} \right)^{2} = 1.2254\left( {\frac{t}{{t_{m} }}} \right)^{{{-}1}} \left\{ {1{-}\exp \left[ {{-}2.3367\left( {\frac{t}{{t_{m} }}} \right)^{2} } \right]} \right\}^{2}$$

As shown in Fig. 3e, although both BLi and SCP@Li are difficult to detect because of their rapid electrochemical reactions at the initial stage prior to *t*_*m*_ [72, 73], BLi and SCP@Li are closer to the 3DI and 3DP nucleation models after *t*_*m*_, respectively. The 3DI nucleation of BLi was characterized by the depletion of a limited number of nucleation sites during the very early nucleation. In addition, because the ideal 3DI model does not consider reactions other than Li deposition, the large deviation between the ideal 3DI model and the dimensionless transient curve of BLi originates from the continuous electrolyte decomposition reaction. This suggests an excessive electrolyte consumption and an unstable SEI of BLi during Li nucleation and growth [73]. By contrast, SCP@Li displayed a *j*_*m*_ (10.57 mA cm^−2^) substantially higher than that of BLi (0.29 mA cm^−2^), attributing to the numerous active sites and high nucleation rate according to Scharifker’s theory (Fig. S21) [71, 74]. And the 3DP model, similar to the Li nucleation behavior of SCP@Li, indicates the progressive activation of nucleation sites accompanied by concurrent nuclei growth. This typically give rise to the formation of smaller particles [75]. The different nucleation modes of BLi and SCP@Li influence the local electrochemical environment, thereby affecting the subsequent growth patterns. Finite element simulations were performed utilizing COMSOL Multiphysics to delve deeper into the post nucleation step. The size and number of the initial Li nuclei on BLi and SCP@Li were determined based on the SEM results. As depicted in Fig. S22, in the initial step (0 s), the current density vectors were focused on the limited nuclei on BLi, whereas those in SCP@Li displayed a high degree of uniform spatial distribution throughout a large number of progressively generated nuclei, yielding a more uniform Li-ion flux. In the Li growth process (Fig. S23), the preferential deposition of Li ions onto pre-existing nuclei exacerbated the non-uniform and localized Li growth behavior in BLi, ultimately leading to the formation of detrimental protrusions (Fig. 3f). However, owing to the homogeneous distribution of the current density and Li-ion flux, the Li nuclei on SCP@Li underwent uniform and planar growth, suppressing excessive volume expansion and protrusions (Fig. 3g).

Based on the aforementioned experimental and simulation results, a Li nucleation and growth mechanism for BLi and SCP@Li is proposed (Fig. 3h). For BLi, the limited number of Li nucleation sites, high Li nucleation barrier, and 3DI nucleation mode yield non-uniform and large Li nuclei within a significantly short timeframe during the initial nucleation process. Subsequently, Li ions are concentrated on these random Li nuclei without additional Li nucleation during the Li growth process. This further intensifies the localized Li deposition and eventually causes severe volume expansion and Li protrusions. Unlike BLi, because the SCP modulation layer has abundant Li nucleation sites, a low Li nucleation barrier, and a 3DP nucleation mode, minute and numerous Li nuclei are generated progressively and uniformly over the initial nucleation process. The effectively modulated Li nuclei ultimately grow into a close-packed Li deposition layer capable of suppressing dendritic growth, volume expansion, and side reactions between the electrolyte and Li deposits. The modulated Li deposition behavior of SCP@Li is the foundation for improving the electrochemical kinetics and reversibility of LMBs.

### Assessing the Electrochemical Characteristics of SCP@Li

**Fig. 4:**
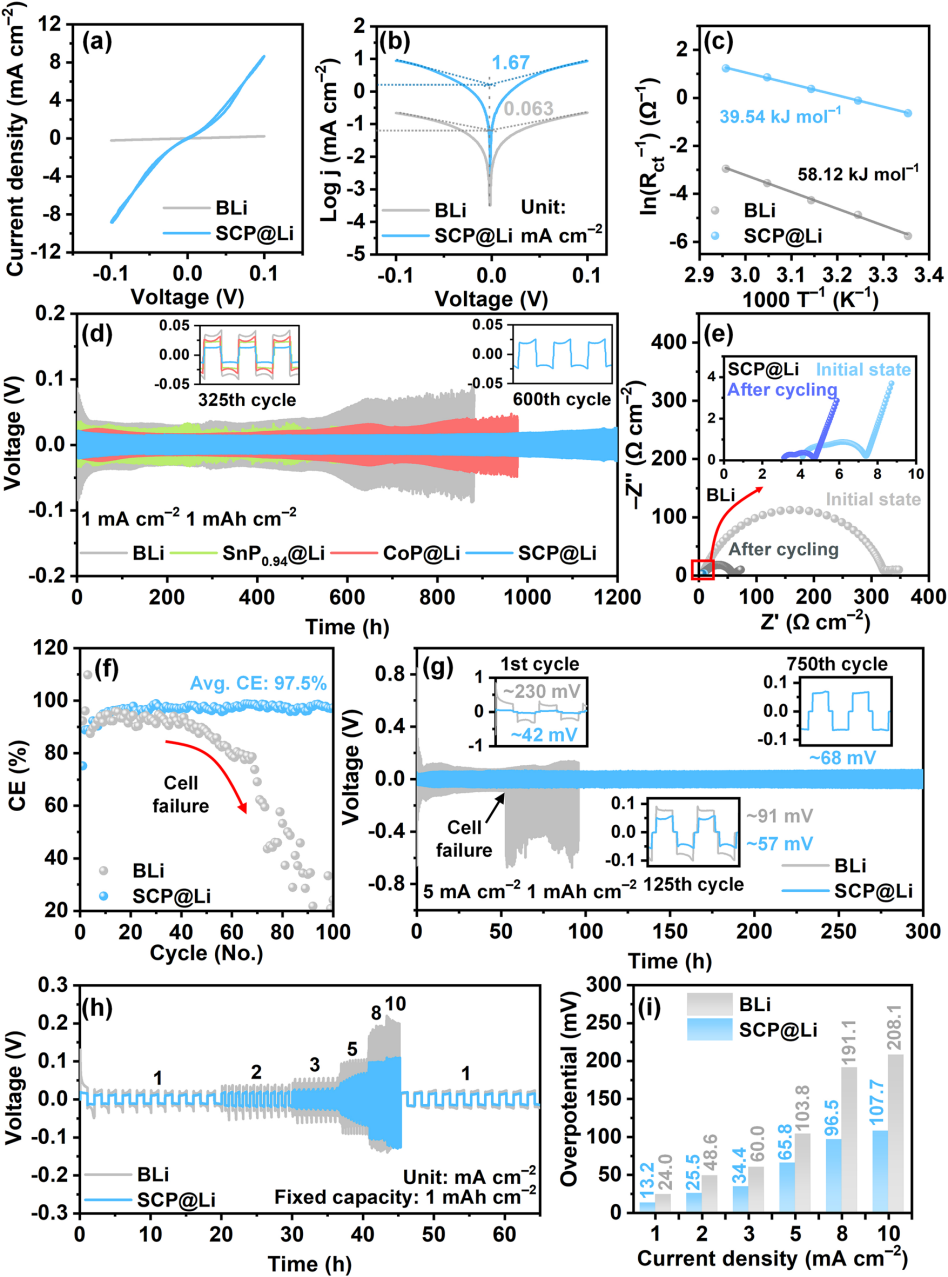
Electrochemical analysis of SCP@Li. **a** CV curves and corresponding **b** Tafel plots of BLi and SCP@Li symmetric cells. **c** Activation energies for Li^+^ desolvation. **d** Voltage–time profiles under a current density of 1 mA cm^−2^ with a plating capacity fixed at 1 mAh cm^−2^. **e** Nyquist plots at initial state and after 10 cycles of BLi and SCP@Li symmetric cells. **f** CEs of Li deposition on BLi and SCP@Li asymmetric cells. **g** Voltage–time profiles under a current density of 5 mA cm^−2^ with a plating capacity of 1 mAh cm^−2^. **h** Rate performance of BLi and SCP@Li symmetric cells. **i** Comparison of overpotentials of BLi and SCP@Li symmetric cells corresponding to Fig. 4h

To investigate the influence of the SCP modulation layer on electrochemical kinetics, various electrochemical characterizations of symmetric and asymmetric cells with BLi and SCP@Li electrodes were performed. Figure 4a shows the typical centrosymmetric loops of cyclic voltammetry (CV) profiles of the BLi and SCP@Li symmetric cells [76]. Furthermore, compared with BLi (0.30 mA cm^−2^), the CV profiles of SCP@Li demonstrate larger current responses (8.41 mA cm^−2^) in the 1st cycle and a higher coincidence in the selected (the 1st, 5th, and 10th) cycles (Fig. S24). This demonstrates the better reversibility of Li plating/stripping and improved interfacial electrochemical reaction kinetics [77]. To investigate the electrochemical reactions during Li plating/stripping of SCP, symmetric CV tests were additionally performed over wider potential windows (Fig. S25). Based on the results of CV tests, the SCP undergoes gradual transformation primarily through a spontaneous reaction with Li metal, leading to the formation of Li-Sn alloy capable of reversible electrochemical reaction. In particular, although the SCP modulation layer can result in gradual modification of the SEI composition during repeated Li plating/stripping, the well-overlapped CV curves of SCP@Li symmetric cell indicate that the formed SEI displays a remarkable excellent electrochemical stability [78]. A similar tendency is observed in the CV curves of the asymmetric Li//Cu cells at a sweep rate of 2 mV s^−1^ (Fig. S26). Typically, the two redox peaks at approximately 0 V in the CV curves indicate Li plating/stripping on the working electrodes [79]. The cell with SCP@Li displays higher peak current densities (stripping peak current density (*J*_peak,strip_): 7.86 mA cm^−2^, plating peak current density (J_peak,plate_): 6.79 mA cm^−2^), and a lower onset potential of lithiation (100 mV, inset of Fig. S26) compared with BLi (J_peak,strip_: 1.70 mA cm^−2^, J_peak,plate_: 1.21 mA cm^−2^, onset potential: 130 mV). This clearly indicates the enhanced electrochemical redox kinetics realized by SCP@Li. The exchange current density (*j*_0_) was measured to compare the reaction kinetics at the electrode/electrolyte interface. If the effects of concentration polarization are insignificant at the interface, j_0_ can be calculated from the Tafel equation according to the CV curves (Fig. 4b) [21]. The j_0_ of the SCP@Li symmetric cell (1.67 mA cm^−2^) is higher than that of the BLi symmetric cell (0.063 mA cm^−2^). This result implies improved charge transfer capability and faster interfacial electrochemical reactivity in the SCP@Li symmetric cell, which is in agreement with the CV results of the symmetric cells [21]. The Li-ion conduction and desolvation was measured via temperature-dependent electrochemical impedance spectroscopy (EIS) using cells with blocking electrodes and Li symmetric cells (Figs. S27 and S28). Prior to the evaluation of Li-ion conduction and desolvation resistance, the ionic conductivity (*σ*) of the cell with the SCP layer (calculated from the EIS spectra at room temperature) exhibited 10.0 mS cm^−1^. This was almost 1.77-fold higher than that of the blank cell (5.65 mS cm^−1^). Also, by linear fitting of the Arrhenius equation, the activation energies (E_a_) of SCP layer were determined as 2.74 and 39.54 kJ mol^−1^ for Li-ion conduction and desolvation, respectively (Figs. S27c and 4c). The high *σ* and low *E*_a_ of the SCP modulation layer verifies the important roles of SCP in accelerating the Li-ion desolvation effect and Li-ion transportation owing to higher Li-ion adsorption energy and BIEF of SCP heterostructure and, thereby, mitigating the adverse effects of increased electrode thickness [80–82].

To validate the long-term durability and reversibility of SCP@Li for LMBs, the galvanostatic cycling performance of the SCP@Li symmetric cells was measured and compared with those of other symmetric cells with BLi, CoP@Li, and SnP_0.94_@Li electrodes. As shown in the voltage–time profiles at a current density of 1 mA cm^−2^ with the plating capacity fixed at 1 mAh cm^−2^ (Fig. 4d), SCP@Li exhibited the lowest overpotential with remarkable cycling stability for ~1200 h. The remarkably reduced overpotential can be indexed to the various advantages of the lithiophilic CoP/SnP_0.94_ heterostructure (including the uniform redistribution of Li ions and charge at the heterointerface, stable SEI formation, and dense Li deposition without dendrites) [47]. Conversely, the BLi and CoP@Li symmetric cells suffered a gradual increased polarization and voltage oscillation after ~525 and ~725 h, respectively. This is attributed to the rapid increase in impedance resulting from the severe side reactions including electrolyte consumption and accumulation of inactive “dead Li” during repeated Li plating/stripping [50]. Unlike the others, the voltage–time profile of SnP_0.94_@Li (Fig. S29) exhibited intense fluctuations followed by a dramatic voltage decrease after ~ 600 h, indicating conventional soft short circuit in Li symmetric cells [83, 84]. As seen in the insets of Fig. 4d, SCP@Li exhibited a marginal overpotential (14.5 mV, 325th cycle). Meanwhile, BLi, CoP@Li, and SnP_0.94_@Li exhibit 41.3 mV, 31.6 mV, and 23.1 mV, respectively. Additionally, the SCP@Li symmetric cell exhibits a gradual polarization over 1200 h without an illusion of long-term cycle performance originating from the hard short-circuit in the cell. This indicates an ultra-slow controlled consumption of active Li and minimal “dead Li” formation during the prolonged cycling [21, 85]. To further investigate the electrochemical reaction kinetics of the assembled symmetric cells, the Nyquist plots of the BLi and SCP@Li symmetric cells was obtained before/after cycling at 1 mA cm^−2^ with 1 mAh cm^−2^ (Fig. 4e and Table S2). By fitting electrochemical resistance values based on the equivalent circuit (Fig. S30), the interfacial resistance (*R*_int_ = the resistance of SEI (*R*_SEI_) + the charge transfer resistance (*R*_ct_)) of the BLi symmetric cell fell from ~307.4 to ~53.7 Ω after 10 cycles, indicating the increased surface area due to porous Li deposits generated during Li plating/stripping [5]. With regard to the SCP@Li symmetric cell, the initial *R*_int_ value (~3.0 Ω) was much smaller than that of the BLi cell, ascribed to elevated electrochemical kinetics and active surface area by introducing the SCP modulation layer [86]. After 10 cycles, the R_int_ value of SCP@Li decreased to ~ 1.54 Ω. This further confirmed the gradual SEI stabilization and the formation of the smooth deposited Li surface without Li dendrites during the initial plating/stripping process. Furthermore, the decreased *R*_bulk_ and *R*_int_ are attributed to the gradual formation of Li_3_P and inorganic-rich SEI (further discussed in the section on SCP transformation after cycling), which is composed of highly Li-ion conductive species [87]. Specifically, at an equal current density, the SCP@Li renders a higher average CE (~97.53%) for 100 cycles. This suggests the high reversibility of Li plating/stripping behavior on the SCP modulation layer. Meanwhile, BLi exhibits remarkable CE drops after 40 cycles (Fig. 4f). We also evaluated the cycling stability of the symmetric cells in a harsher current density of 5 mA cm^−2^ with fixed capacity of 1 mA cm^−2^. As shown in Fig. 4g, the SCP@Li symmetric cell maintained a low overpotential (~68 mV) for up to 300 h (= 750th cycle) with a significantly flat voltage plateau. However, the voltage–time profiles of the BLi symmetric cell exhibited intense voltage fluctuations throughout the cycles, which intensified after 53 h (= 132th cycle) owing to uncontrollable Li dendrite growth. The superior cycling performance of SCP@Li was compared with previously reported surface-modified LMAs, as summarized in Table S3. To validate the reversibility of the symmetric cells under variable current densities, we conducted rate tests on the BLi and SCP@Li symmetric cells at a fixed capacity of 1 mAh cm^−2^ (Fig. 4h). The sequential implementation of current densities of 1, 2, 3, 5, 8, and 10 mA cm^−2^ yielded stair-shaped voltage–time profiles. This is indicative of corresponding stepwise increased overpotentials. The cells using SCP@Li/BLi presented overpotentials of 13.2/24.0, 25.5/48.6, 34.4/60.0, 65.8/103.8, 96.5/191.1, and 107.7/208.1 mV, respectively (Fig. 4i). Here, SCP@Li demonstrated a significantly lower overpotential at each stage and an approximately two-fold lower overpotential than BLi at the maximal current density of 10 mA cm^−2^. The key reason for the remarkable rate performance of the SCP@Li symmetric cell is likely to be the combination of a high rate of diffusion during Li-ion dissolution and the rapid Li-ion conductivity at the electrolyte/SCP@Li interface [28]. In contrast, the BLi symmetric cell sustained irreversible critical damage under increased current densities (≥ 8 mA cm^−2^). This was inferred from the observation of rapidly increasing overpotentials. Moreover, when the current density decreased to 1 mA cm^−2^, the overpotential (~15.8 mV) in the SCP@Li symmetric cell returned to be similar values, indicating unobstructed Li-ion transfer pathways within the electrode and its outstanding cycling reversibility and durability.

### Monitoring the Chemical Transformation of SCP and Morphological Evolution in SCP@Li after Cycling

**Fig. 5:**
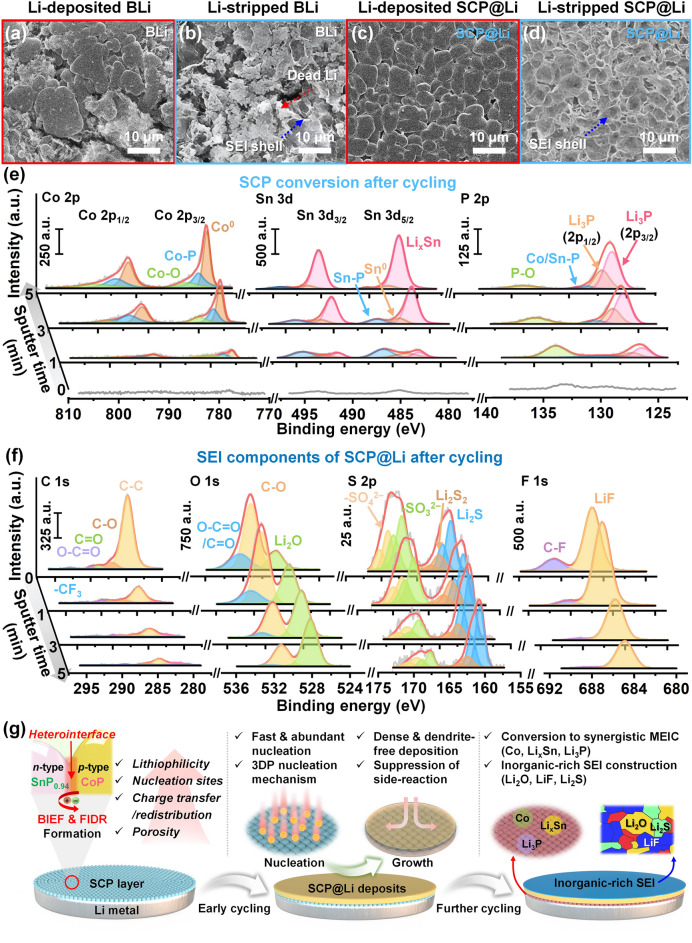
Analysis of morphology and chemical composition after cycling. SEM images of **a** Li-deposited BLi, **b** Li-stripped BLi, **c** Li-deposited SCP@Li, and **d** Li-stripped SCP@Li after 50 cycles at a current density of 1 mA cm^−2^ under a capacity of 1 mAh cm^−2^. **e** Depth-profiled XPS spectra of SCP modulation layer in SCP@Li after cycling. **f** Depth-profiled XPS spectra of SEI on SCP@Li after cycling. **g** Schematic illustration of comprehensive Li deposition mechanism during cycling of SCP modulation layer

To reveal the effect of SCP modulation layer, the morphological evolution of BLi and SCP@Li was investigated after 50 cycles (100 h) at a current density of 1 mA cm^−2^ under an areal capacity of 1 mAh cm^−2^. The high (Fig. 5a) and low (Fig. S31a) magnification SEM images of the Li-deposited BLi surface show loosely connected, non-uniform deposits with inconsistent sizes, as well as significantly accumulated lumps of pulverized “dead Li” and broken SEI layers. The Li-stripped BLi surface (Figs. 5b and S32a) further revealed deleterious lumps, in conjunction SEI shells originating from the dissolution of reversible Li deposits. The surface morphology of the cycled BLi demonstrates the non-uniform, porous Li deposition behavior and the resulting irreversibility of BLi. This irreversibility deteriorates the lifetime and electrochemical kinetics of LMBs during subsequent cycling owing to the rapid electrolyte and fresh Li consumption and an increased resistive fraction [88]. In contrast, the Li-deposited SCP@Li (Figs. 5c and S31b) still displays compact and dendrite-free Li deposition layer consisting of relatively homogeneous “cell-like” Li deposits over a broad area. In particular, as shown in Figs. 5d and S32b, the key distinction between Li-stripped SCP@Li and BLi is that SCP@Li exhibits only SEI shells with a morphology identical to that of Li deposits observed on the Li-deposited SCP@Li without inactive Li accumulation and destroyed SEI. This indicates a reversible Li plating/stripping within the SEI shells analogous to the breathing process of the lungs [89]. These observations also emphasize the important role of the SCP modulation layer in enhancing reversibility and eliminating malicious inactive components.

Beyond optimizing Li deposition, the variation in the chemical composition of the SCP modulation layer and the SEI component evolution of SCP@Li after cycling were investigated. Before cycling, the XRD patterns (Fig. S33) and XPS spectra (Fig. S34a–c) of SCP@Li verified the retention of the pristine SCP powders. To further understand the spontaneous reaction between SCP and Li metal, XPS depth profiling analysis was performed on pristine SCP@Li and a thinner SCP layer-coated Li. As shown in Fig. S34d, even after sufficient Ar^+^ sputtering for 30 min, pristine SCP@Li exhibited no significant difference from the P 2*p* XPS spectrum before depth profiling, without any trace of Li_3_P, the conversion product of CoP and SnP_0.94_. For SCP in close contact with Li (thin coating layer), a small fraction of Li_3_P was observed along with transition metal phosphide bonding (Fig. S34e). The existence of Li compound between SCP layer and Li metal is also evidenced in Li 1*s* spectrum (Fig. S34f). This XPS depth profiling analysis indicates that although some conversion reaction occurred in the lower layer in contact with Li before cycling, the original SCP composition was well maintained throughout the SCP layer. After 50 cycles at a current density of 1 mA cm^−2^ under a capacity of 1 mAh cm^−2^, the disappearance of the SCP crystalline phase and the observation of only metallic Li peaks in the XRD pattern of SCP@Li indicate the transformation of SCP into an amorphous phase (Fig. S33). XPS depth profiling analysis was employed to examine the transformation of the SCP during cycling (Fig. 5e). Prolonged sputtering revealed distinct peaks corresponding to the constituent elements of the SCP. After 5 min of Ar^+^ sputtering, the cycled SCP@Li showed a significant reduction in the intensity of the peaks associated with the pristine SCP (Fig. 1h–j). Concurrently, new peaks corresponding to Co^0^ (791.5/775.87 eV) [90], Li_*x*_Sn (490.3/481.9 eV) [91], and Li_3_P (127.2/126.3 eV) [92] became dominant in the Co 2*p*, Sn 3*d*, and P 2*p* XPS spectra. The conversion of CoP and SnP_0.94_ to metallic Co, Li_*x*_Sn alloy, and Li_3_P is attributed to the strong reducing potential of metallic Li, which ultimately resulted in compositional variations within the SCP [41]. Because the SCP modulation layer was designed to maintain its function as an MEIC that can simultaneously transport Li ions and electrons, unlike electron-insulating layers or purely electron-conductive layers, even with compositional variations during cycling, SCP@Li can stably induce Li plating/stripping processes over extended cycles by synergistically incorporating metallic Co (high electronic conductivity), Li-Sn alloy (high Li affinity) [93], and Li_3_P (high ionic conductivity). The introduction of an SCP modulation layer can also influence the SEI by regulating its composition during long-term cycling and adapting the environment to promote efficient Li plating/stripping [94]. Thus, the SEI components of SCP@Li and BLi after cycling were evaluated further via XPS depth profiling (Figs. 5f and S35). In terms of C 1*s* XPS spectra, before Ar^+^ sputtering, both cycled BLi and SCP@Li mainly revealed organic components including C–O (286.5 eV, originated from DOL/DME solvent decomposition or remnants), C=O and O–C=O (288.1 and 289.7 eV, respectively, originated from the decomposition products of the solvents), and –CF_3_ (292.8 eV, originated from the decomposition or remnants of TFSI^−^ anion) [95]. However, with an increase in Ar^+^ sputtering time, the intensity of the organic species reduced gradually within the cycled BLi and SCP@Li. Meanwhile, SCP@Li exhibited a significantly lower proportion of organic species (particularly C=O and O–C=O) than BLi (Fig. S36). Moreover, the newly appearing C-Li peak (282.5 eV) of SCP@Li indicates the likely presence of metallic Li after Ar^+^ sputtering. This implies that the SEI thickness was thinner than the BLi [96]. Similar to the trend of the C 1*s* spectra, with the increase in Ar^+^ sputtering time, the O 1*s* and S 2*p* spectra of SCP@Li also demonstrate a gradual decrease in organic species corresponding to C–O (531.2 eV), C=O/O–C=O (532.3 eV), –SO_4_^2−^ (2*p*_1/2_: 170.2 eV, 2*p*_3/2_: 169.1 eV), and –SO_3_^2−^ (2*p*_1/2_: 168.3 eV, 2*p*_3/2_: 167.1 eV) that is more rapid than that of BLi. Significantly, the inner SEI of SCP@Li is predominantly composed of inorganic Li_2_O (528.5 eV), Li_2_S_2_ (2*p*_1/2_: 163.1 eV eV, 2*p*_3/2_: 161.9 eV), and Li_2_S (2*p*_1/2_: 161.4 eV eV, 2*p*_3/2_: 160.2 eV), rather than organic species [95, 96]. For the F 1*s* spectra, two typical peaks corresponding to C-F (688.5 eV, originated from LiTFSI remnants) and LiF (685.1 eV, originated from the decomposition of LiTFSI) are observed [97]. Unlike BLi, where the LiF peak maintains a high intensity notwithstanding the increase in etching depth, the reducing LiF peak intensity in SCP@Li with progressive etching depth indicates that the altered dense Li deposition behavior of SCP@Li can suppress the decomposition of LiTFSI. Based on an XPS depth profiling analysis, the calculated atomic proportions of SEIs on SCP@Li and BLi further strengthen that SCP@Li facilitates the formation of an inorganic-rich SEI, unlike BLi (Fig. S37) [98, 99]. These inorganic components provide various advantages. First, the abundance of Li_2_O plays a crucial role in the development of compact SEI [100]. Second, Li_*x*_S components can facilitate enhanced Li-ion diffusion and uniform distribution of Li-ion flux, whereas LiF can increase the mechanical stability and lower the diffusion barrier [101, 102].

Synthesizing these observations, the Li deposition mechanism of the SCP modulation layer is summarized comprehensively (Fig. 5g). During the early Li plating/stripping process, owing to the favorable heterostructure (lithiophilic heterointerface, abundant nucleation sites and enhanced charge redistribution) of the SCP, Li nucleation and growth can be significantly uniform and compact without Li dendrites and protrusions while suppressing side reactions with the electrolyte. As successive cycles proceed, the spontaneous conversion of SCP into Co, Li_*x*_Sn, and Li_3_P composites induced by the spontaneous reaction with metallic Li and the construction of an inorganic-rich SEI play crucial roles in maintaining a stable Li plating/stripping process over long-term cycles.

### Full Cell Performance Evaluation of SCP@Li

**Fig. 6:**
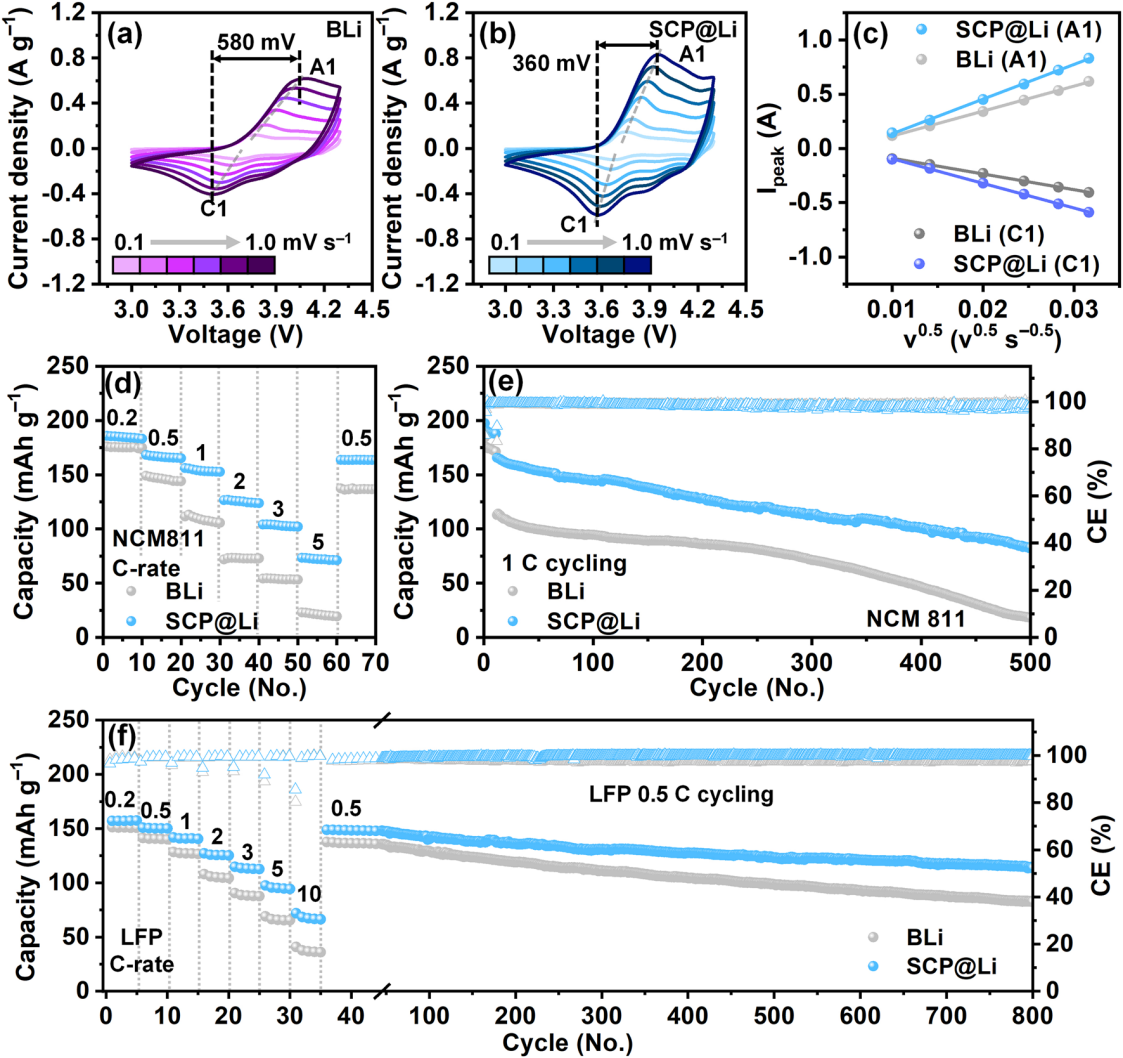
Full cell performance evaluation of SCP@Li. CV curves of **a** NCM811//BLi and **b** NCM811//SCP@Li across the scan rates, and **c** the corresponding linear fit of the cathodic/anodic peak current intensities as a function of the square root of the scan rates. **d** Rate performance and **e** cycling performance of NCM811//BLi and NCM811//SCP@Li cells. **f** Rate performance and subsequent cycling performance of LFP//BLi and LFP//SCP@Li cells

To assess the practical application of SCP@Li in full cells, we examined the feasibility of incorporating SCP@Li into full cells combined with NCM811 cathodes. The CV curves across scan rates (0.1–1.0 mV s^−1^) revealed that the NCM811//SCP@Li cell exhibits more obvious anodic (A1) and cathodic (C1) peaks with higher redox current densities and smaller voltage separation between A1 and C1 peaks (360 mV for NCM811//SCP@Li cell and 580 mV for NCM811//BLi cell) than the NCM811//BLi cell (Fig. 6a, b). This reflects the reduced polarization and enhanced electrochemical reactivity of NCM811//SCP@Li cell. Furthermore, the Li-ion transfer kinetics were analyzed based on the Li-ion diffusion coefficients (${\text{D}}_{{\text{Li}}^{+}}$) using the Randles–Sevcik equation. As shown in Fig. 6c, the A1 and C1 peaks at various scan rates exhibit a linearly proportional relationship with the square root of the voltage scan rates. This indicates a diffusion-controlled electrochemical process in the full cells. The calculated ${\text{D}}_{{\text{Li}}^{+}}$ values of the NCM811//SCP@Li cell for the A1 and C1 peaks were 2.29 × 10^−8^ and 1.16 × 10^−8^ cm^2^ s^−1^, respectively. These are higher than those of NCM811//BLi cell (A1 peak: 1.19 × 10^−8^ cm^2^ s^−1^, C1 Peak: 4.71 × 10^−9^ cm^2^ s^−1^). The result agrees with the outstanding electrochemical kinetics, thereby demonstrating an accelerated Li-ion desolvation effect and enhanced Li-ion transportation, as discussed in Fig. 3.

The rate performances of the NCM811//SCP@Li and NCM811//BLi cells were measured at various C-rates (Fig. 6d). Apparently, with stepwise C-rates from 0.2 C to 5 C, the specific discharge capacities of the NCM811//SCP@Li are 184.8, 166.5, 153.7, 125.6, 103.4, and 72.2 mAh g^−1^, respectively. These distinctly surpass the capacities measured for the NCM811//BLi cell (175.3, 146.5, 108.9, 73.2, 53.9, and 21.3 mAh g^−1^). In addition, NCM811//SCP@Li retained its original specific capacity as the C-rate returned to 0.5 C again. Moreover, the galvanostatic profiles of the NCM811//SCP@Li cell clearly demonstrate overpotentials less than those of NCM811/BLi cell (Fig. S38), indicating the improved rate capability and redox kinetics across current densities. Regarding durability, the NCM811//SCP@Li full cell displayed a higher initial capacity (165.7 mAh g^−1^) and excellent capacity retention (~49.3%), outperforming those of the NCM811//BLi full cell (initial capacity: 114.2 mAh g^−1^, capacity retention: ~15.9%) after 500 cycles at 1 C (Fig. 6e). To further demonstrate the potential of SCP@Li for practical use, LFP full cells with SCP@Li and BLi anodes were subjected to cycling tests combined with C-rate and subsequent long-term galvanostatic cycling. As anticipated (Fig. 6f and Table S4), the LFP//SCP@Li cell displayed improved rate capability and long-term cycling stability (initial capacity: 148.9 mAh g^−1^, capacity retention: ~75.8%) at 0.5 C compared with the LFP//BLi cell (initial capacity: 137.7 mAh g^−1^, capacity retention: ~59.1%).

## Conclusion

To summarize, we proposed a pioneering design for a modulation layer composed of a highly lithiophilic CoP/SnP_0.94_ heterostructure applying the Kirkendall effect. During the initial cycling, the spontaneous FIDR and BIEF formation of the *p–n* junction in the SCP provided abundant lithiophilic nucleation sites and enhanced charge transfer, as demonstrated by experiments and theoretical calculations. This induced a uniform Li nucleation/growth behavior with the suppression of volume expansion, Li dendrite formation, and electrolyte consumption. As revealed by XPS depth profiling analysis, with continuous cycling, the progressive transformation of SCP into the MEIC layer (consisting of metallic Co, lithiophilic Li-Sn alloy, and high ionic conductive Li_3_P) and development of inorganic-rich SEI layer enables the initial stable Li deposition behavior of SCP@Li to persist for long-term cycling. As result, SCP@Li demonstrated superior electrochemical kinetics and performance, including a high ionic conductivity, high exchange current density, and remarkable cycling stability for 750 cycles while sustaining a low overpotential of 68 mV in the symmetric cell test, even at a high current density of 5 mA cm^−2^. Moreover, the LFP//SCP@Li full cell delivered a high-capacity retention of 75.8% for 800 cycles at 0.5 C. This work has revealed the regulatory role of lithiophilic heterostructures on Li deposition behavior and the role of compositional transition in artificial SEI during cycling. This provides a meaningful reference for designing of highly effective modulation layers for LMAs.

## Supplementary Information

Below is the link to the electronic supplementary material.

Supplementary file1 (DOCX 9092 kb)
