# Supplementary material for: Designing Metal Phosphide Solid-Electrolyte Interphase for Stable Lithium Metal Batteries Through Electrified Interface Optimization and Synergistic Conversion
Source: Nanomicro Lett. 2025 Jun 27;17:315. doi: 10.1007/s40820-025-01813-1 (PMC12204979; doi:10.1007/s40820-025-01813-1)
Supplement: Supplementary file 1 — Supplementary file1 (DOCX 9092 kb) [file 40820_2025_1813_MOESM1_ESM.docx]

Supporting Information for

**Designing Metal Phosphide Solid-Electrolyte Interphase for Stable Lithium Metal Batteries through Electrified Interface Optimization and Synergistic Conversion**

Jung Been Park^1#^, Changhoon Choi^2#^, Min Sang Kim^1^, Hyeongbeom Kang^1^, Eunji Kwon^3^, Seungho Yu^3,4^, and Dong-Wan Kim^1^*

School of Civil, Environmental, and Architectural Engineering, Korea University, Seoul 02841, South Korea

Department of Materials Science and Engineering, SungShin Women’s University, Seoul, 01133, South Korea

Energy Storage Research Center, Korea Institute of Science and Technology, 5, Hwarang-ro 14-gil, Seongbuk-gu, Seoul 02792, Republic of Korea

^4^ Division of Energy & Environment Technology, KIST School, Korea University of Science and Technology, Seoul 02792, Republic of Korea

*^#^* Jung Been Park and Changhoon Choi contributed equally to this work.

*Corresponding author. E-mail: [dwkim1@korea.ac.kr](mailto:dwkim1@korea.ac.kr) (Dong-Wan Kim)

**Supplementary Figures and Tables**

**
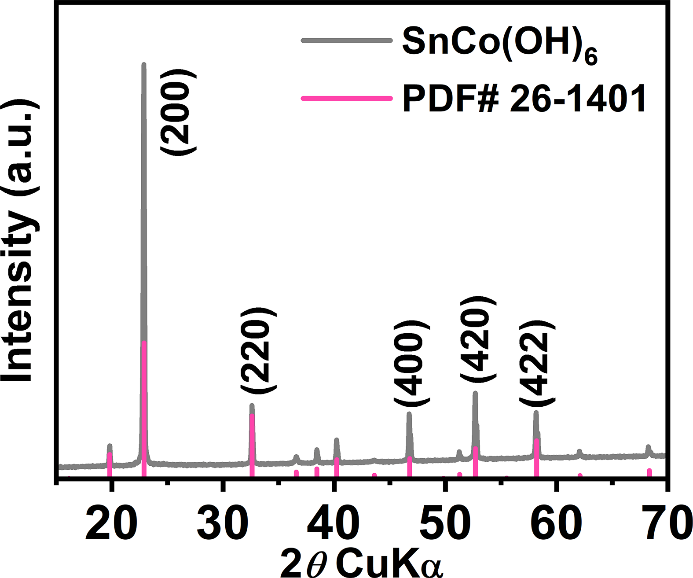
**

**Fig. S1** XRD pattern of SnCo(OH)_6_


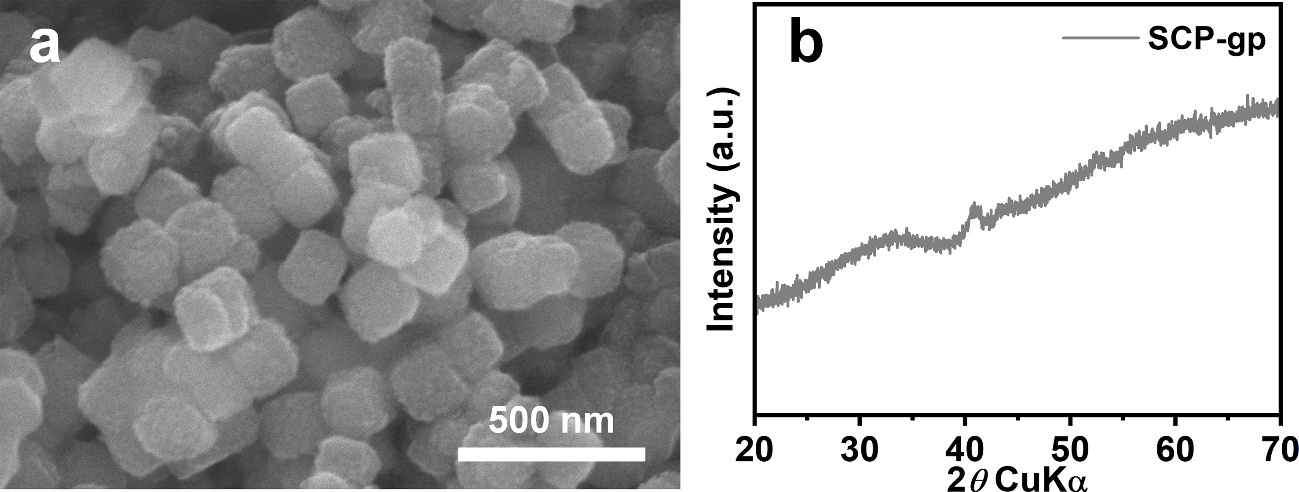


**Fig. S2** **a** SEM image and **b** XRD pattern of SCP-gp

**
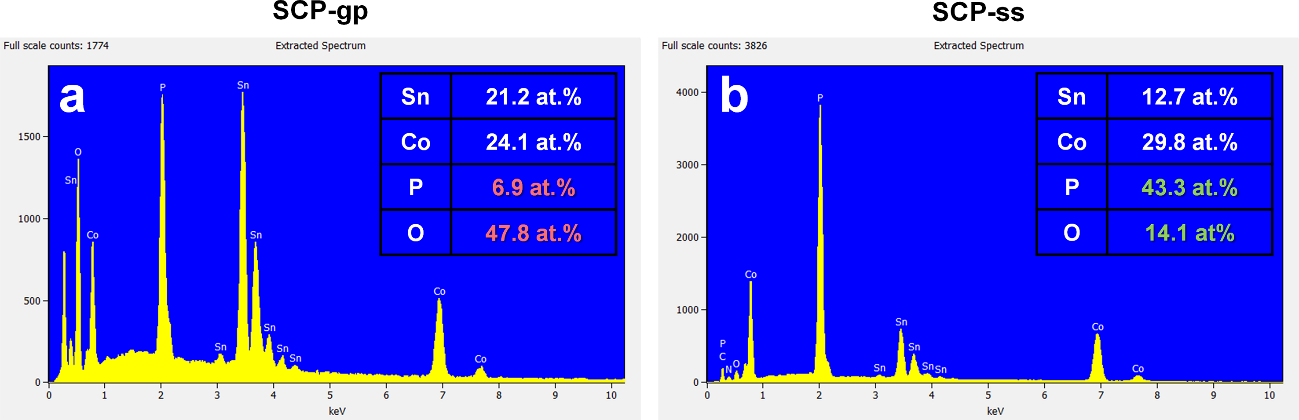
Fig. S3** EDS spectra of **a** SCP-gp and **b** SCP-ss


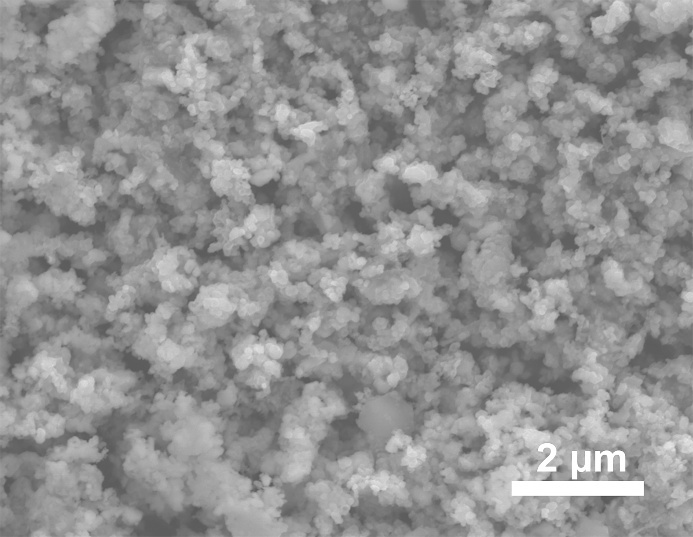


**Fig. S4** Low-magnified SEM image of SCP-ss

**
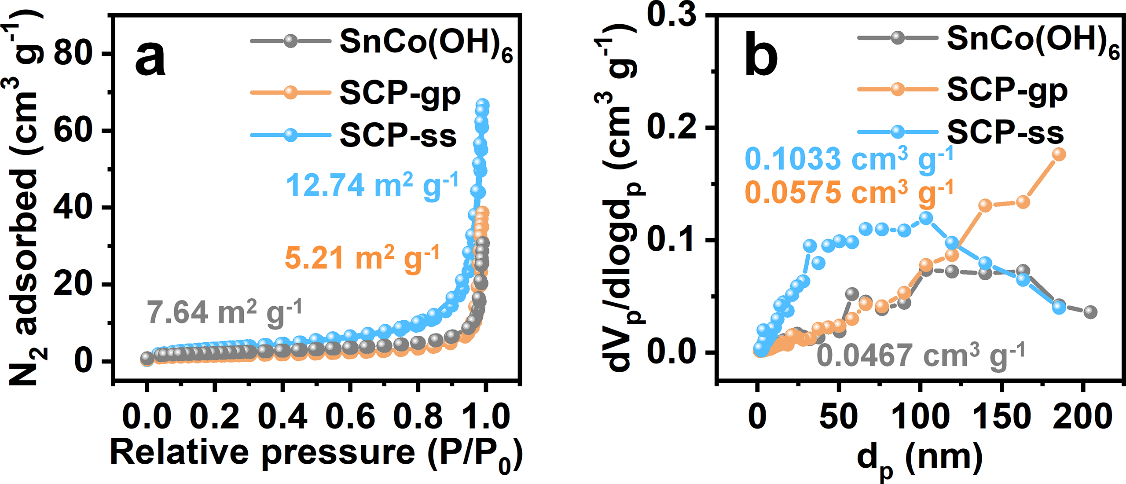
**

**Fig. S5** **a** BET surface area and **b** BJH pore size distribution of SnCo(OH)_6_, SCP-gp, and SCP-ss

**Fig. S6** ICP-OES measurements of the contents Sn, Co, and P in the SCP
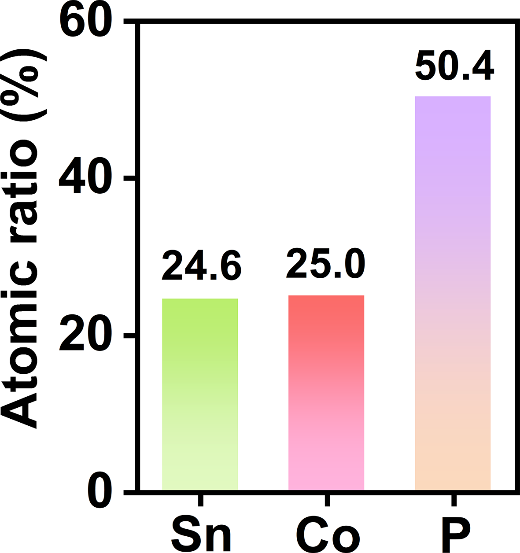


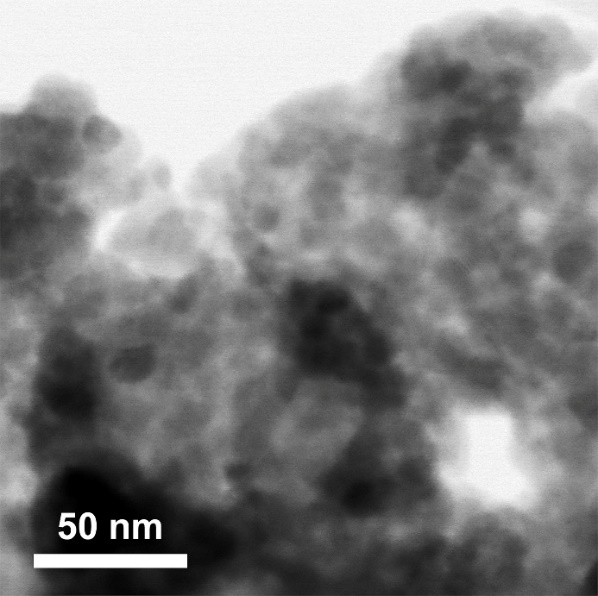


**Fig. S7** High-magnified TEM image corresponding to Fig. 1d.

**
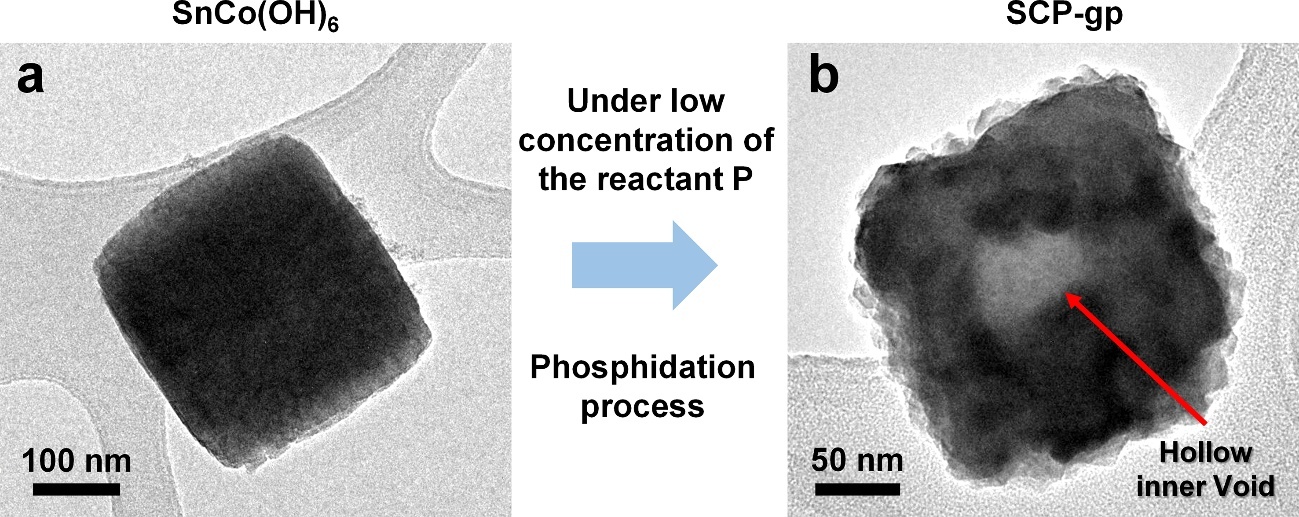
Fig. S8** TEM images of **a** SnCo(OH)_6_ and **b** SCP-gp


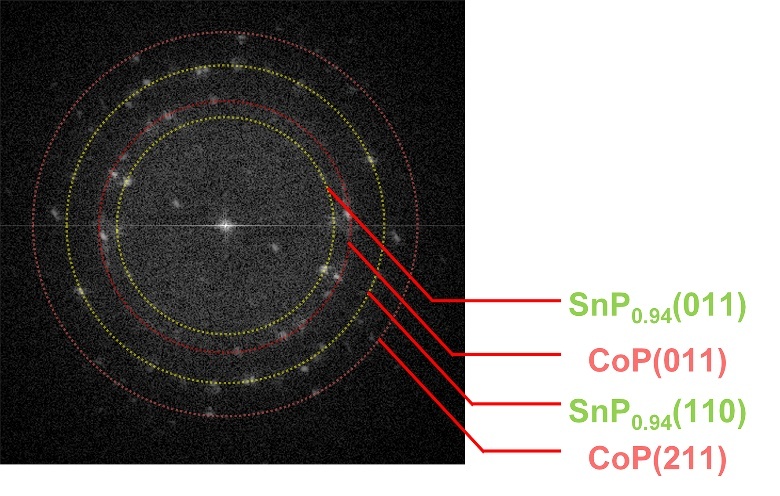


**Fig. S9** FFT pattern corresponding to Fig. 1g

**
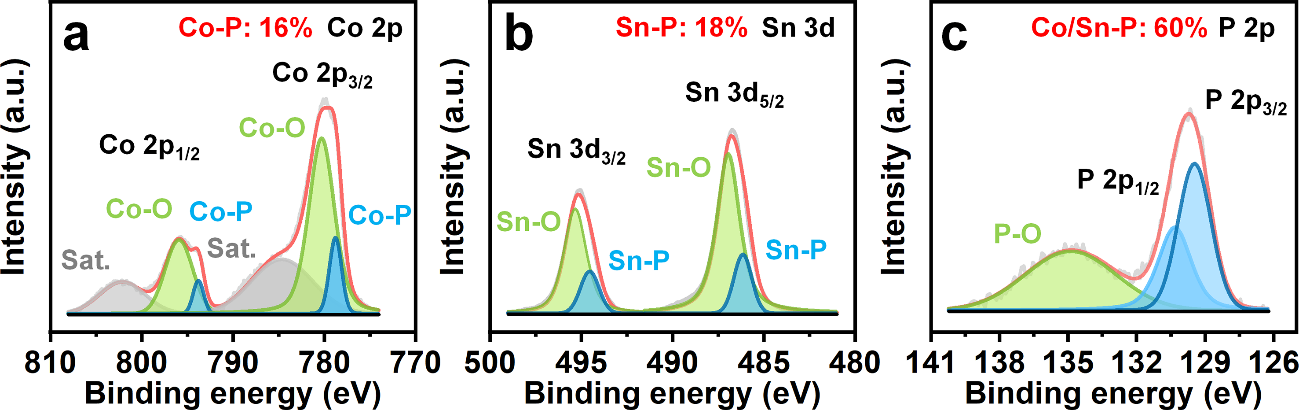
Fig. S10** High resolution XPS spectra of SCP-gp. **a** Co 2p, **b** Sn 3d, and **c** P 2p

**
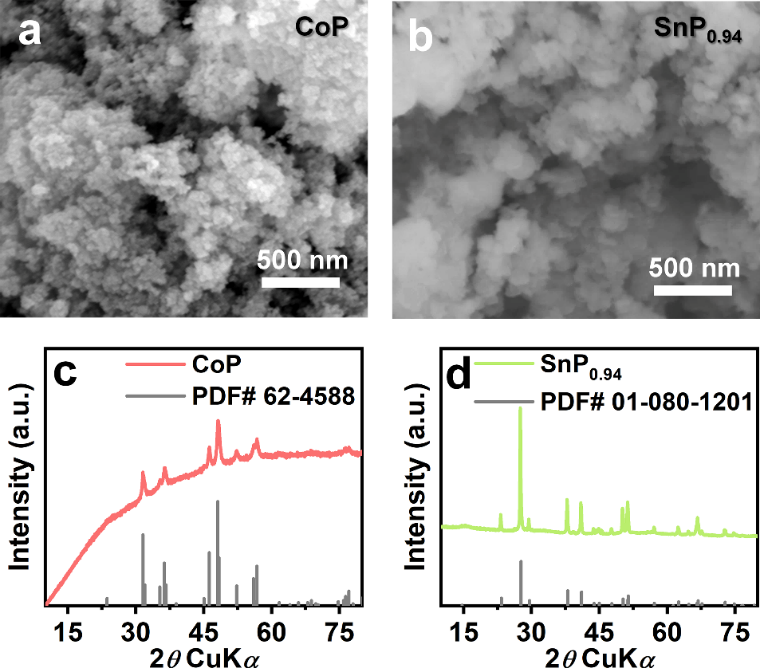
**

**Fig. S11** SEM images of **a** CoP and **b** SnP_0.94_ and corresponding to XRD patterns of **c** CoP and **d** SnP_0.94_


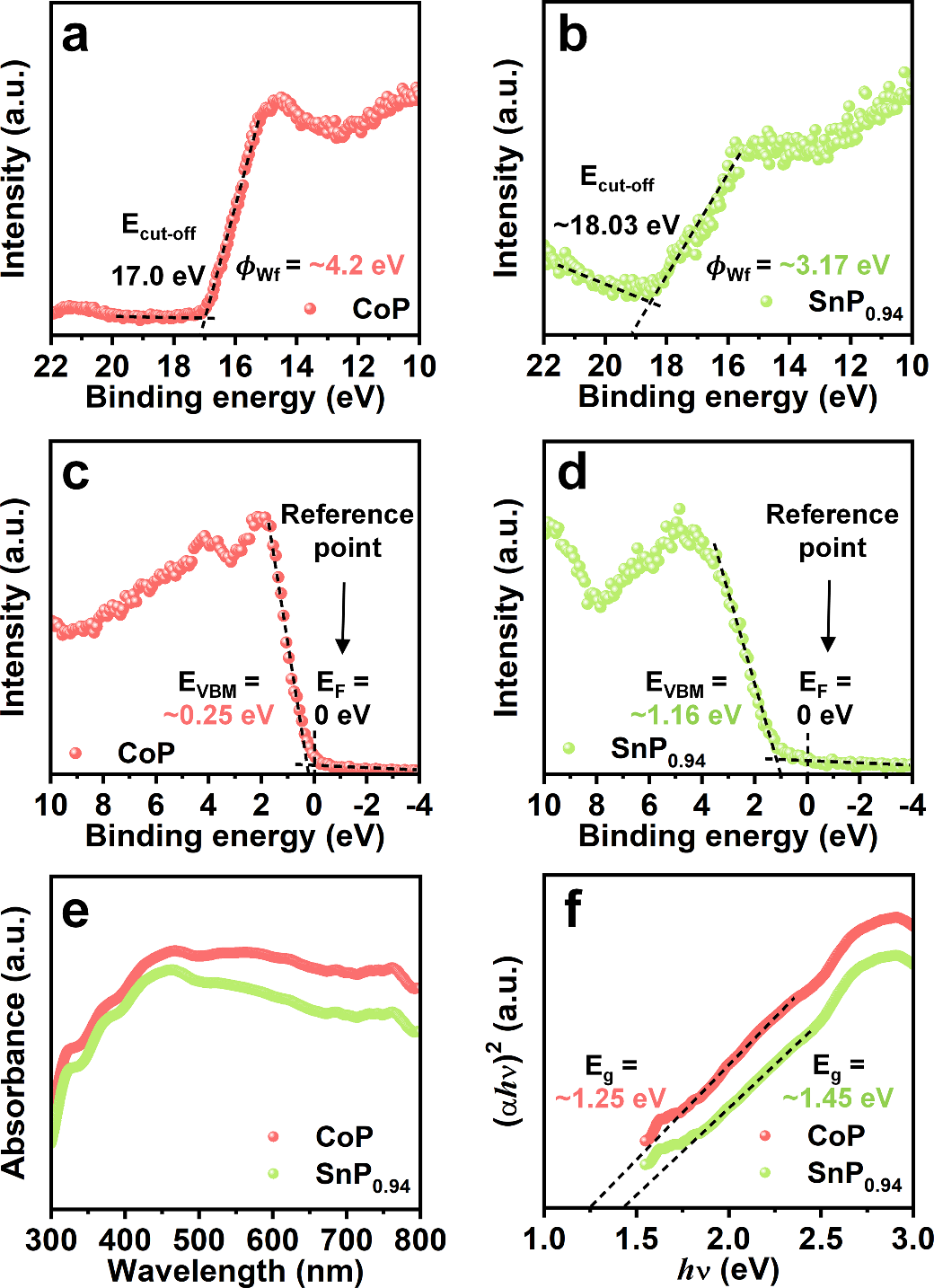


**Fig. S12** UPS spectra of **a** CoP and **b** SnP_0.94_. XPS valence band spectra of **c** CoP and **d** SnP_0.94_. **e** UV-vis absorption spectra of CoP and SnP_0.94_ and **f** corresponding to Tauc plot

<Note>

The work function (*ϕ_Wf_*) is defined as the minimum energy required to remove an electron from the solid surface to a vacuum level (E_vac_–E_F_). Thus, the *ϕ_Wf_* of materials can determine E_F_. By integrating the difference between E_VBM_ and E_F_ with the E_g_ (conduction band minimum energy level (E_c_) – valence band maximum energy level (E_v_)) and *ϕ_Wf_*, we can determine the position of E_F_ within the band gap for each material.


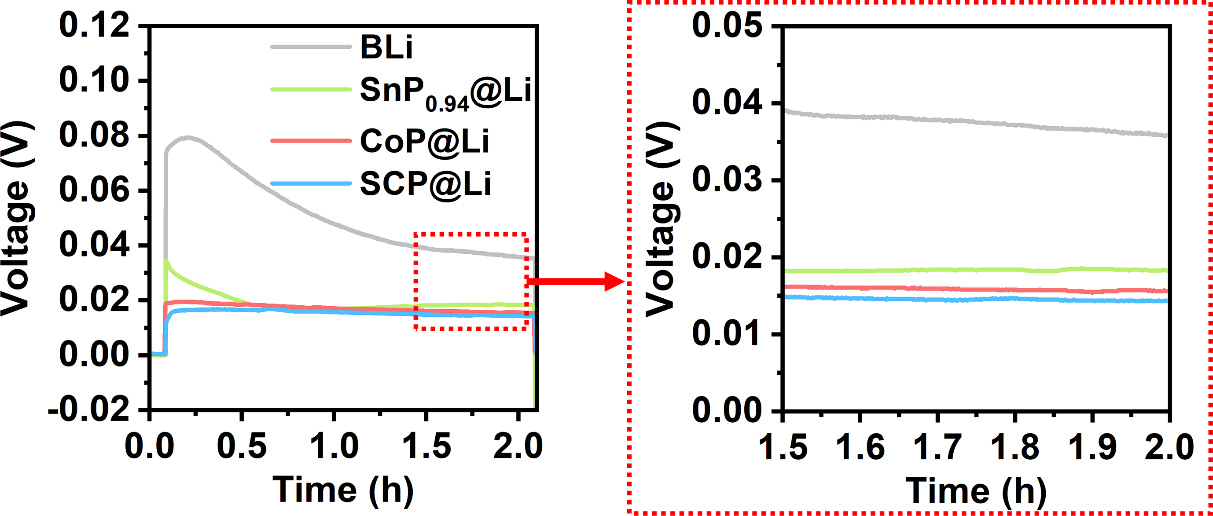


**Fig. S13** Voltage-time profiles of BLi, SnP_0.94_@Li, CoP@Li, and SCP@Li at a current density of 0.5 mA cm^–2^


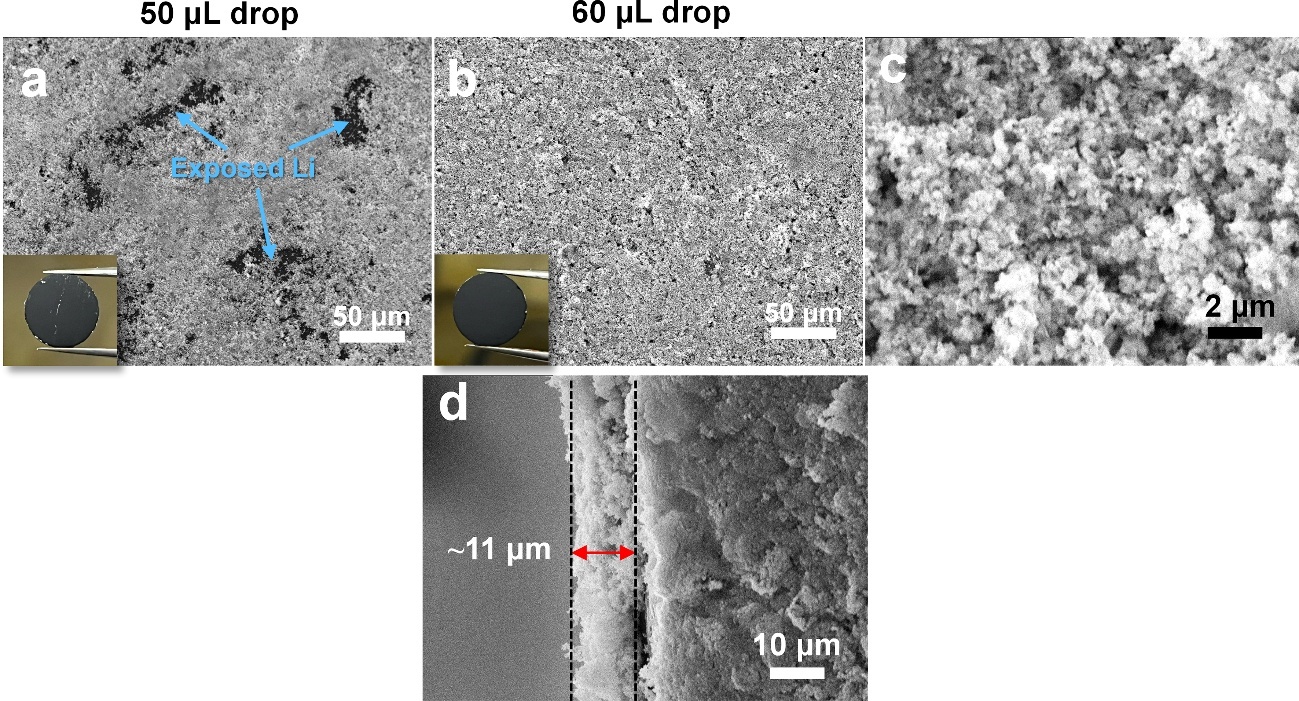


**Fig. S14** SEM and digital images of SCP@Li after drop casting using **a** 50 uL volume and **b** 60 uL volume. **c** high magnified SEM images corresponding to Fig. 14b. **d** Cross-sectional SEM image of optimized SCP@Li.

<Note>

Due to the complex interactions between the solvent, particles, and substrate that influence layer quality, exposure of Li metal in localized areas was unavoidable during drop casting at a 50 µL volume. Therefore, to ensure a stable and uniform coating on the entire Li surface, we optimized drop casting for those conditions (SCP: 20 mg, solvent (toluene): 1 mL, drop volume: 60 μL, Li foil: 15 Pi).


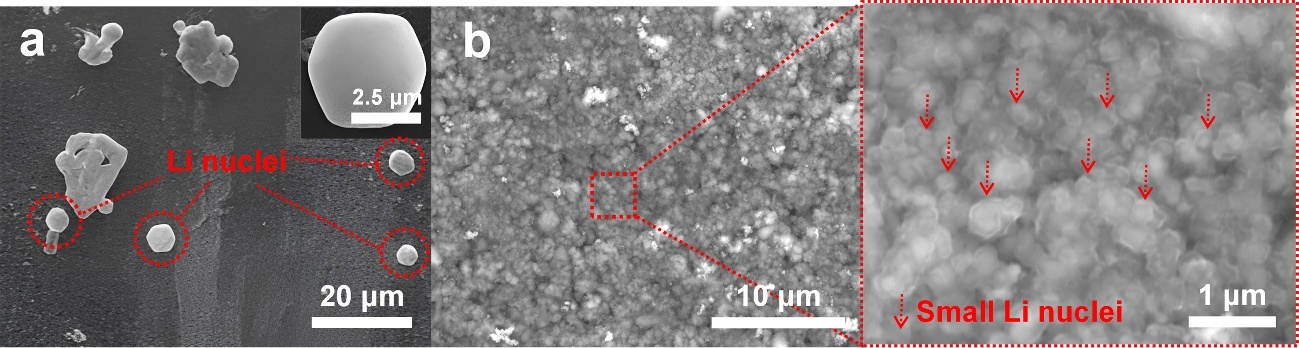


**Fig. S15** SEM images of **a** BLi and **b** SCP@Li after Li deposition of 0.05 mAh cm^–2^


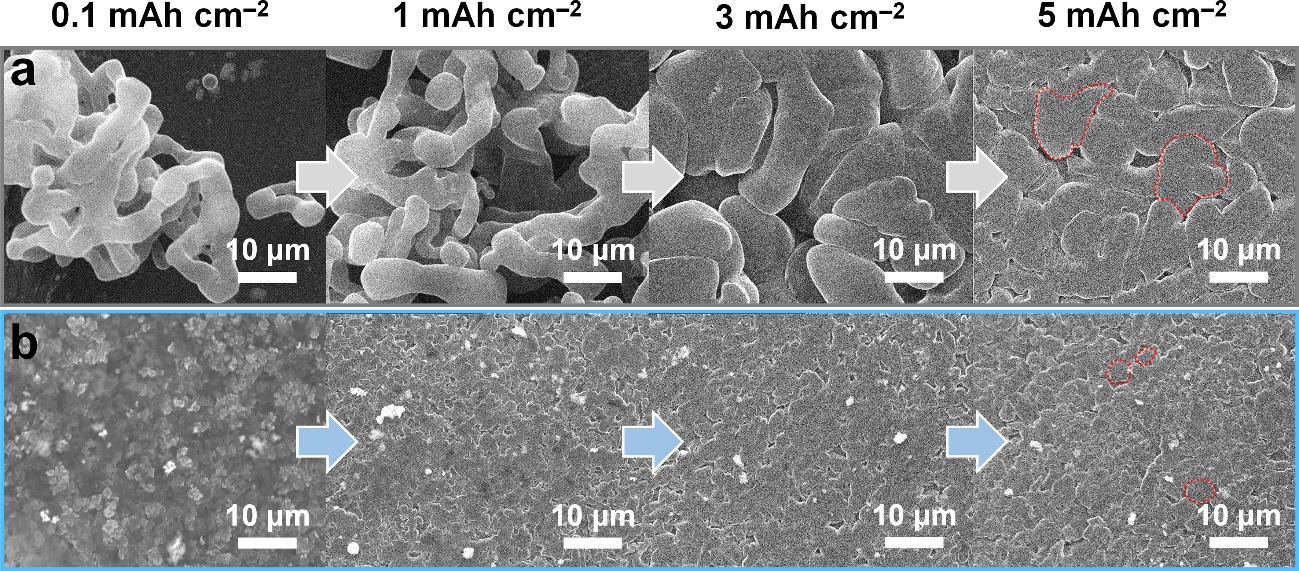


**Fig. S16** The enlarged ex-situ SEM images of **a** BLi and **b** SCP@Li corresponding to Fig. 3a, b


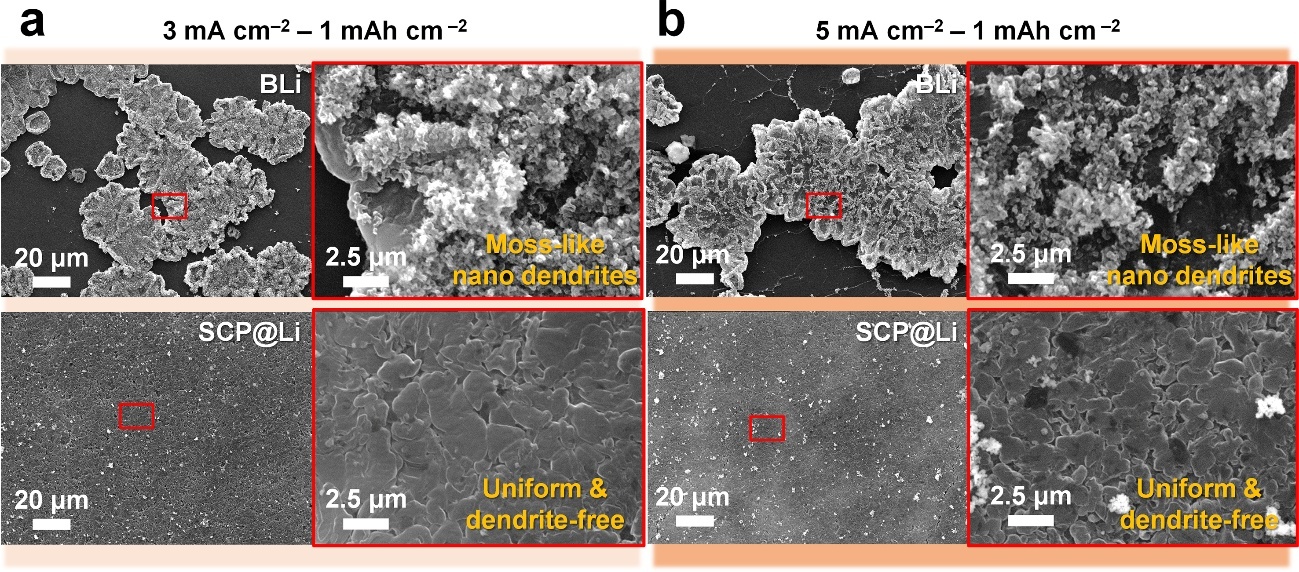


**Fig. S17** Ex-situ SEM images of BLi and SCP@Li after Li deposition. (**a**) A current density of 3 mA cm^–2^ under an areal capacity of 1 mAh cm^–2^. (**b**) A current density of 5 mA cm^–2^ under an areal capacity of 1 mAh cm^–2^

**Fig. S18** Galvanostatic profiles of BLi and SCP@Li at a current density of 3 mA cm^–2^
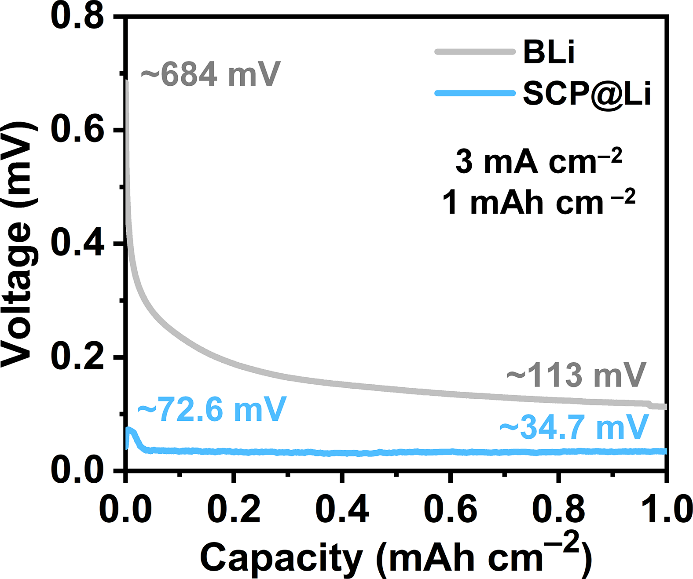


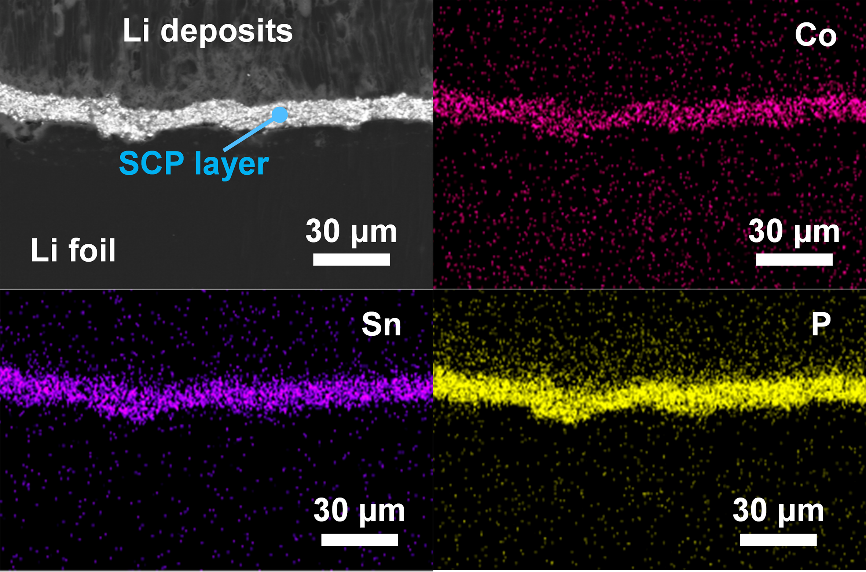


**Fig. S19** BSE and EDS elemental mapping images of SCP modulation layer after Li deposition of 5 mAh cm^–2^


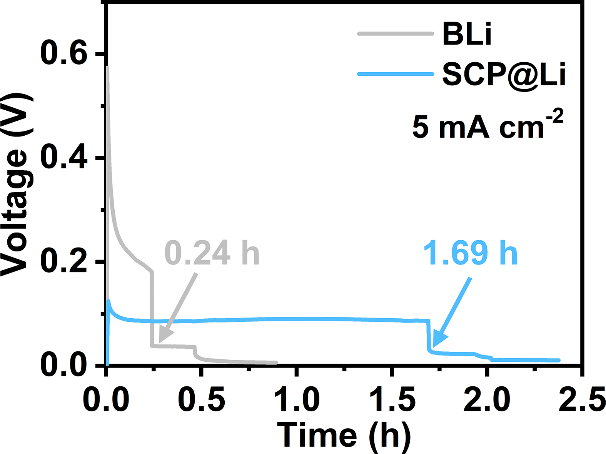


**Fig. S20** Constant current polarization test for BLi and SCP@Li at a current density of 5 mA cm^–2^


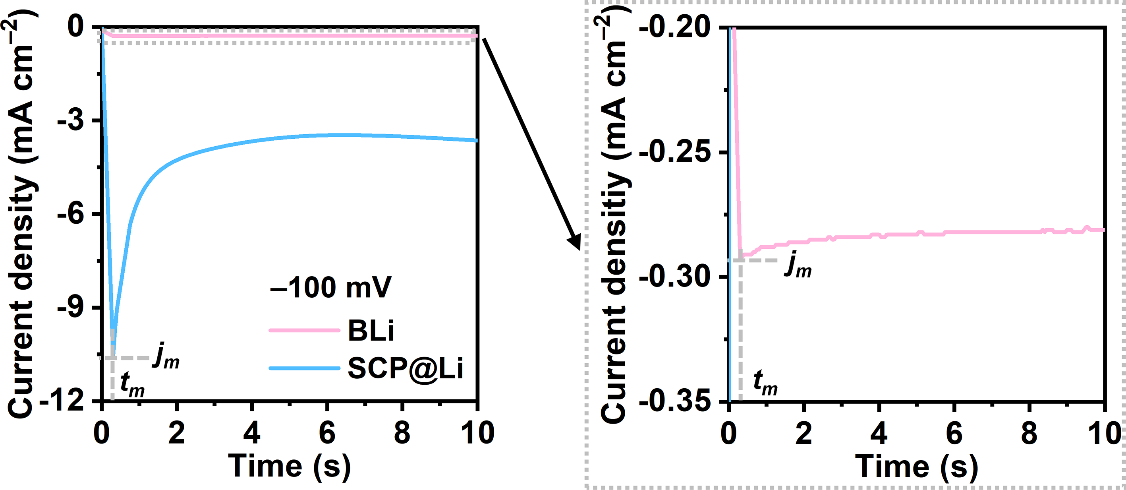


**Fig. S21** Current-time transients obtained at a potential of –100 mV

**<Note>**

B.R. Scharifker’s theory follows the Equation S1 below.

$I = zFc\left( \frac{D}{\pi t} \right)^{\frac{1}{2}}\left( 1-exp\left\{ -N_{0}\pi kD\left[ t-\frac{1-e^{-At}}{A} \right] \right\} \right)$ (S1-1)

$k = \left( \frac{8\pi cM}{\rho} \right)^{\frac{1}{2}}$ (S1-2)

where $I$ is the current density, $zF$, $D$, and $c$ denote the molar charge of the electro-plating species, diffusion coefficient, and bulk concentration of the electrolyte, respectively. $M$ and $\rho$ are the molecular mass and the density of deposit, respectively. $A$ and $N_{0}$ represent the nucleation rate constant and quantity of active sites on the electrode, respectively. The large number of active sites and low nucleation barrier in SCP@Li compared with BLi results in an increased overall current density, as depicted in Fig. S21.


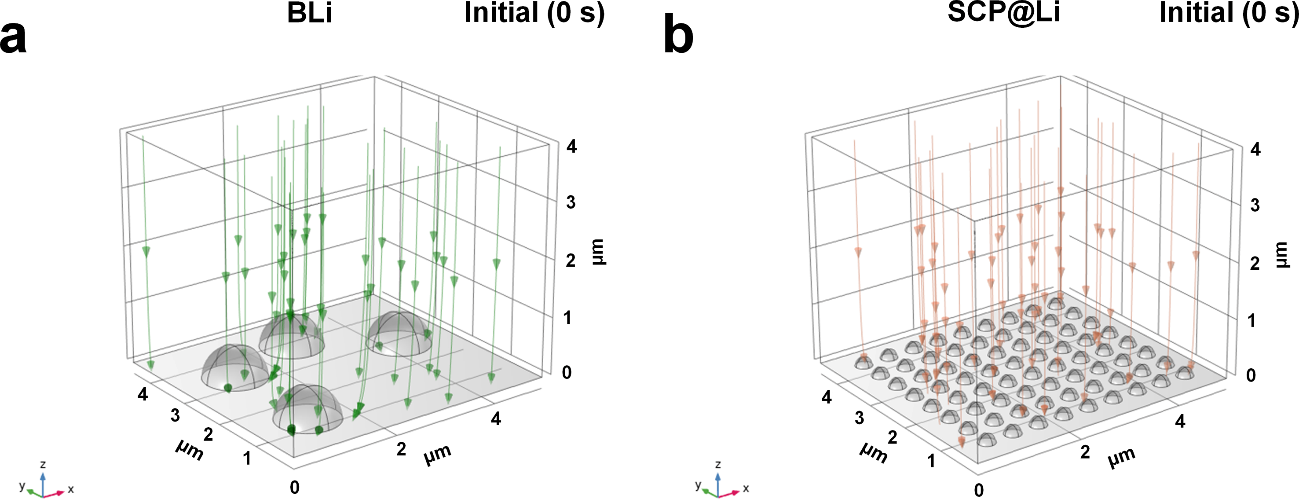


**Fig. S22** The current density vectors on **a** BLi and **b** SCP@Li at initial step (0 s) obtained by numerical simulation


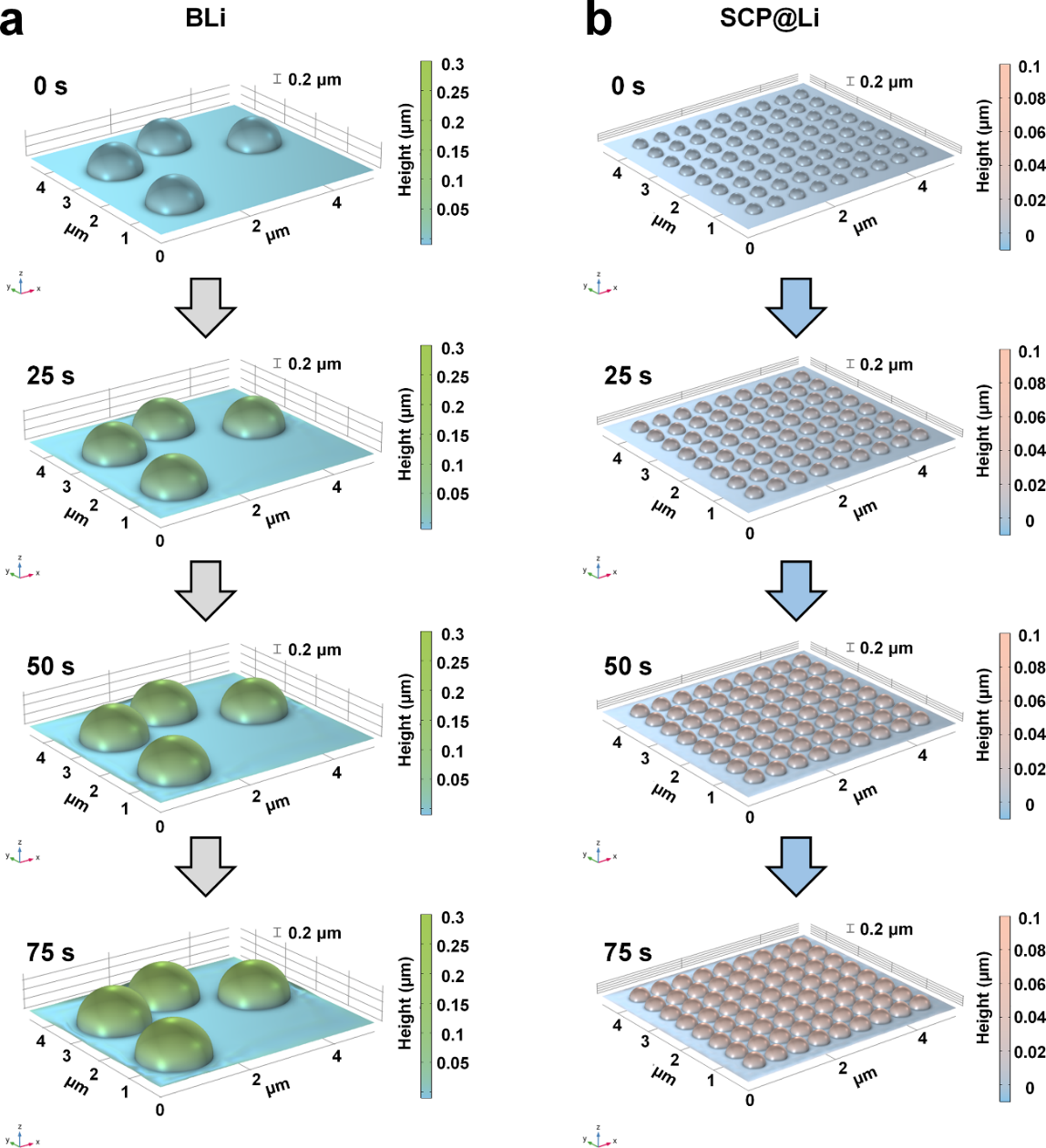


**Fig. S23** The stepwise growth of Li nuclei on **a** BLi and **b** SCP@Li

**
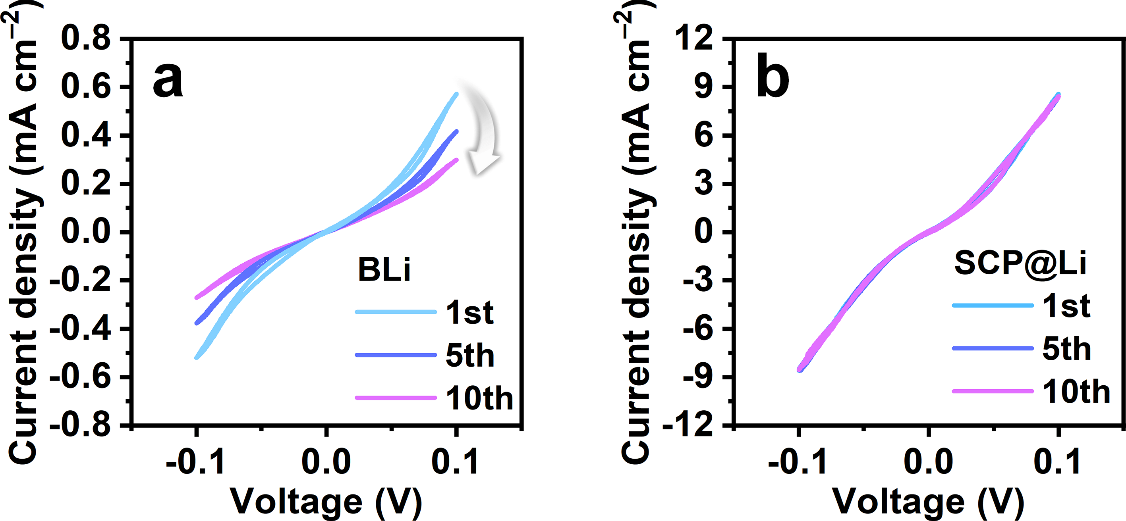
**

**Fig. S24** CV curves of **a** BLi and **b** SCP@Li symmetric cells at the 1st, 5th, and 10th cycle


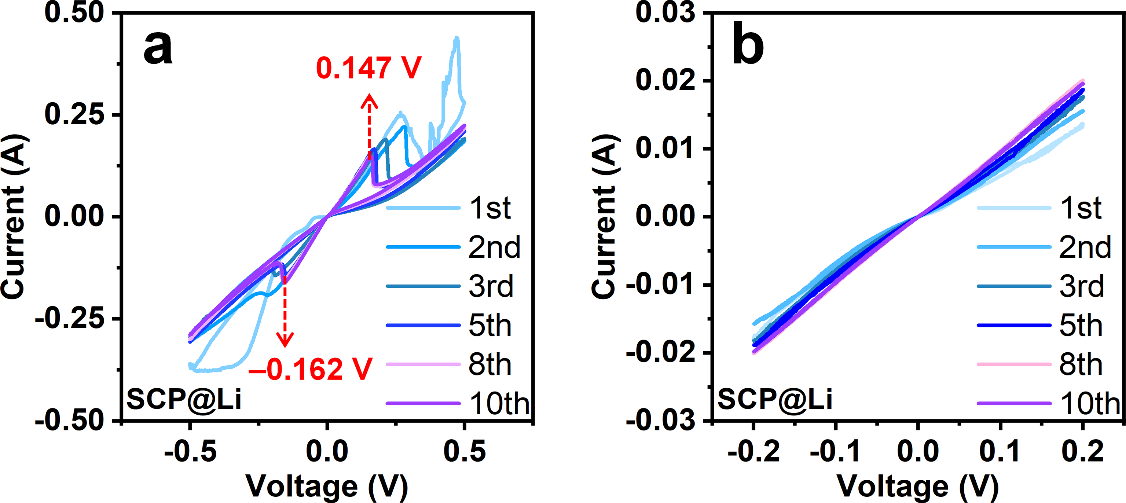


**Fig. S25** CV curve of SCP@Li symmetric cell in the two potential windows (**a** –0.5 to +0.5 V and **b** –0.2 to +0.2 V) at a scan rate of 0.2 mV s^–1^

<Note>

As shown in Fig. S25a, after the first cycle (SCP conversion reaction), Li-Sn alloy/dealloy peaks in SCP@Li is clearly reversible around ~0.15 V. However, when the SCP did not undergo electrochemical lithiation reaction in the first cycle (Fig. 25b), Li-Sn alloy/dealloy peaks are not clearly observed. Based on the results of CV tests, if most of the SCP initially undergoes electrochemical lithiation, leading to sufficient Li-Sn alloy formation, the Li-Sn alloy/dealloy reaction would occur prominently and reversibly during subsequent Li plating/stripping. However, when the potential for SCP electrochemical lithiation is not reached during continuous Li plating/stripping, the Li-Sn alloy/dealloy reaction does not appear prominently.


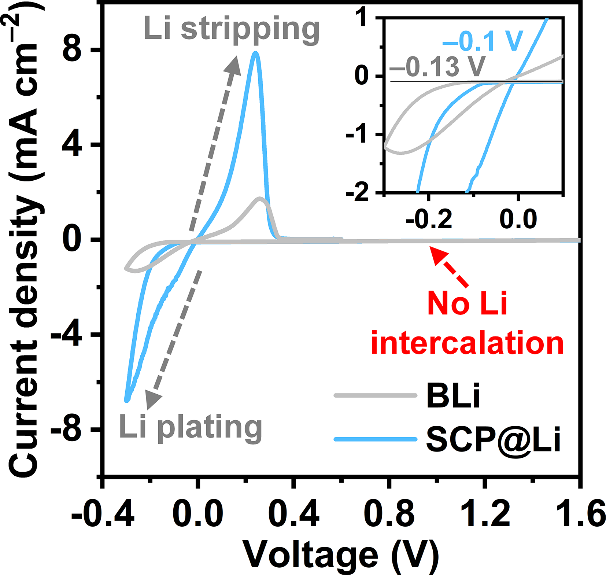


**Fig. S26** CV curves of **a** BLi and **b** SCP@Li asymmetric cells

**
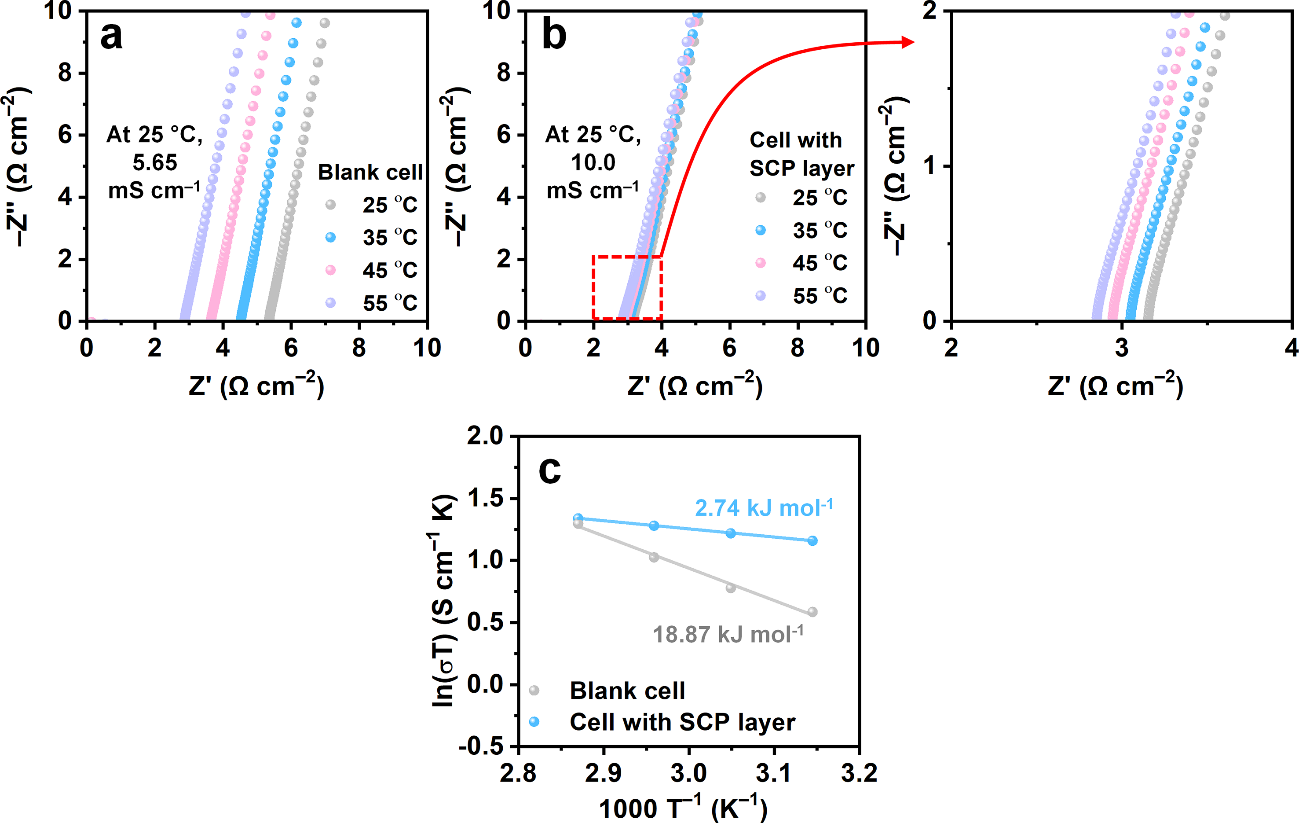
**

**Fig. S27** Nyquist plots of **a** blank cell and **b** cell with SCP layer at different temperatures. **c** The activation energies for Li^+^ conduction

**
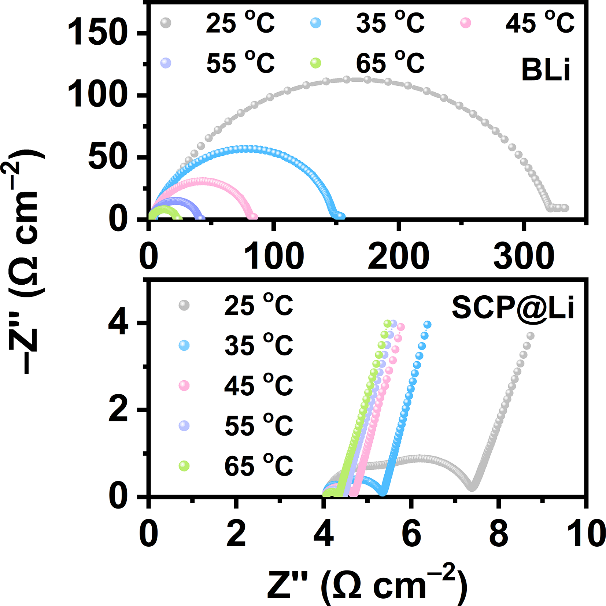
**

**Fig. S28** **a** Nyquist plots of BLi and SCP@Li symmetric cell at different temperatures.

**
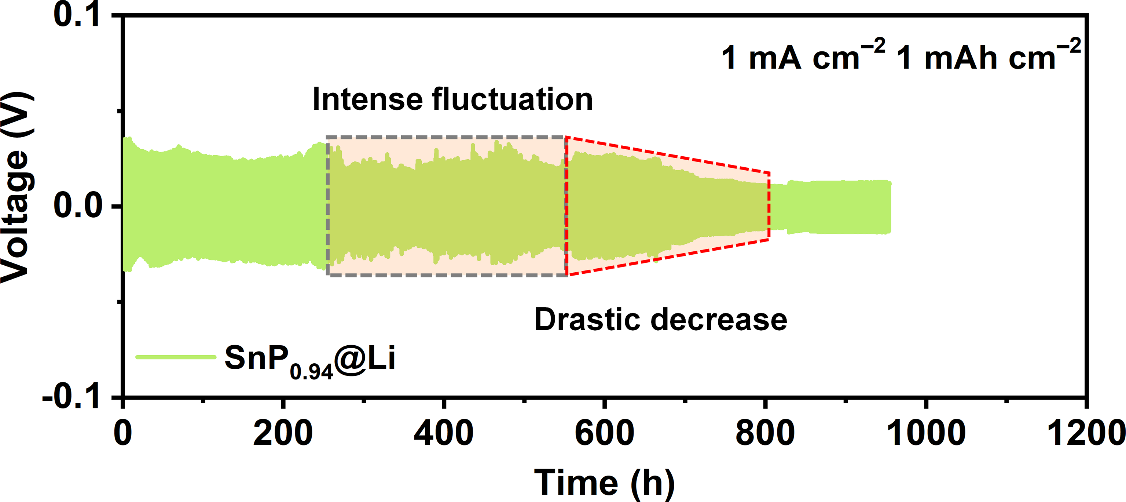
**

**Fig. S29** Voltage-time profiles of SnP_0.94_@Li symmetric cell





**Fig. S30** The equivalent circuit model for Nyquist plots of BLi and SCP@Li symmetric cells corresponding to Fig. 4e


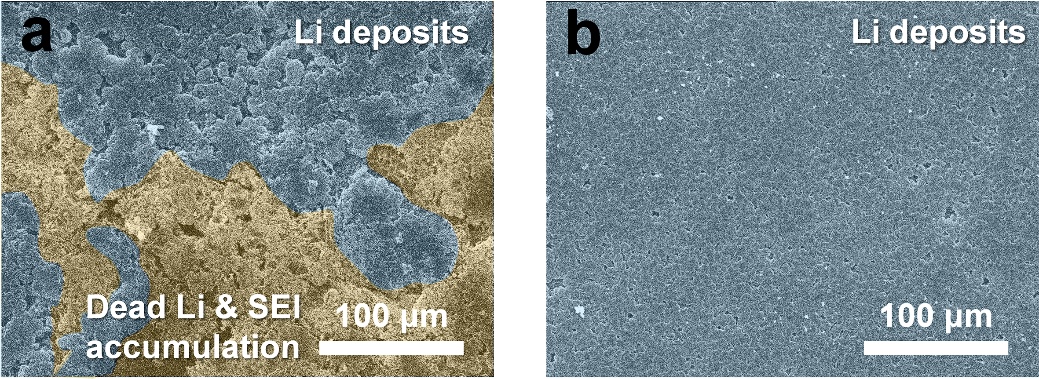


**Fig. S31** Low magnification SEM images of **a** Li-deposited BLi and **b** Li-deposited SCP@Li after 50 cycles at a current density of 1 mA cm^–2^ with an areal capacity of 1 mAh cm^–2^


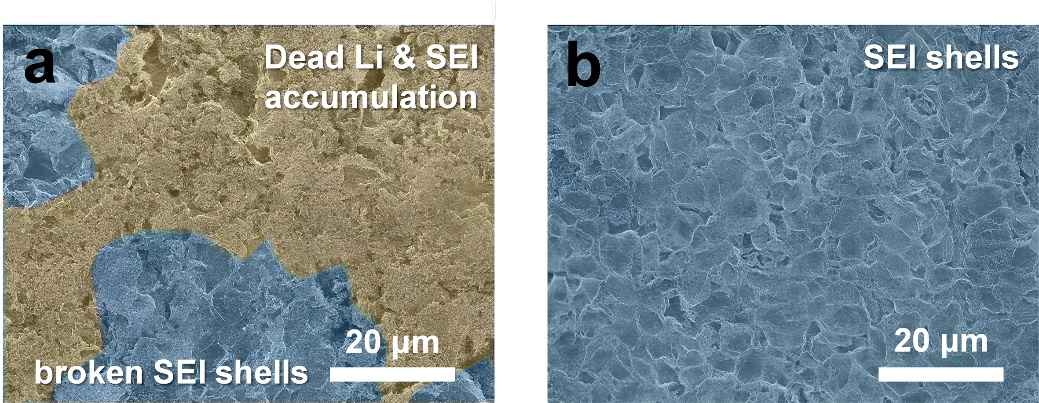


**Fig. S32** Low magnification SEM images of **a** Li-stripped BLi and **b** Li-stripped SCP@Li after 50 cycles at a current density of 1 mA cm^–2^ with an areal capacity of 1 mAh cm^–2^


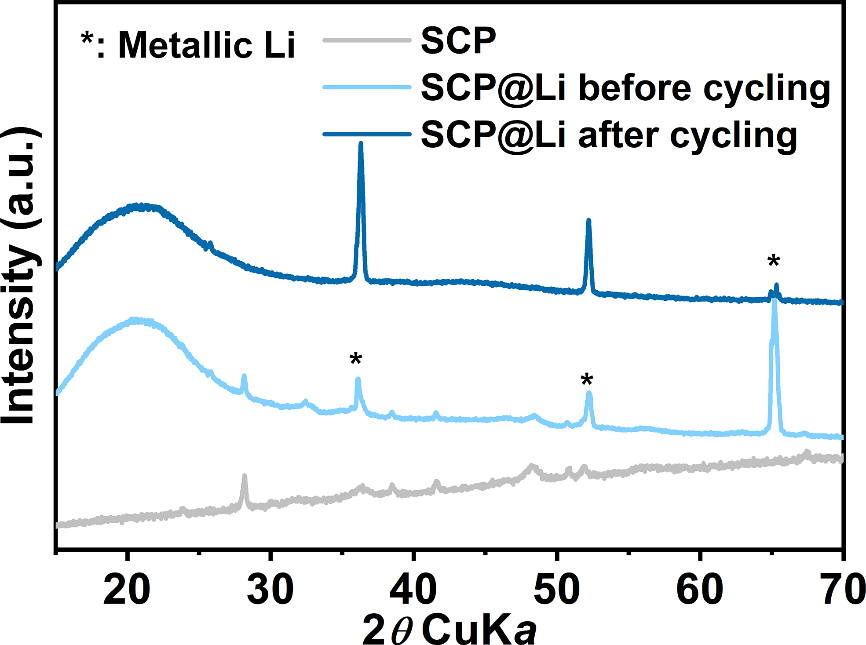


**Fig. S33** XRD patterns of SCP and SCP@Li before and after cycling


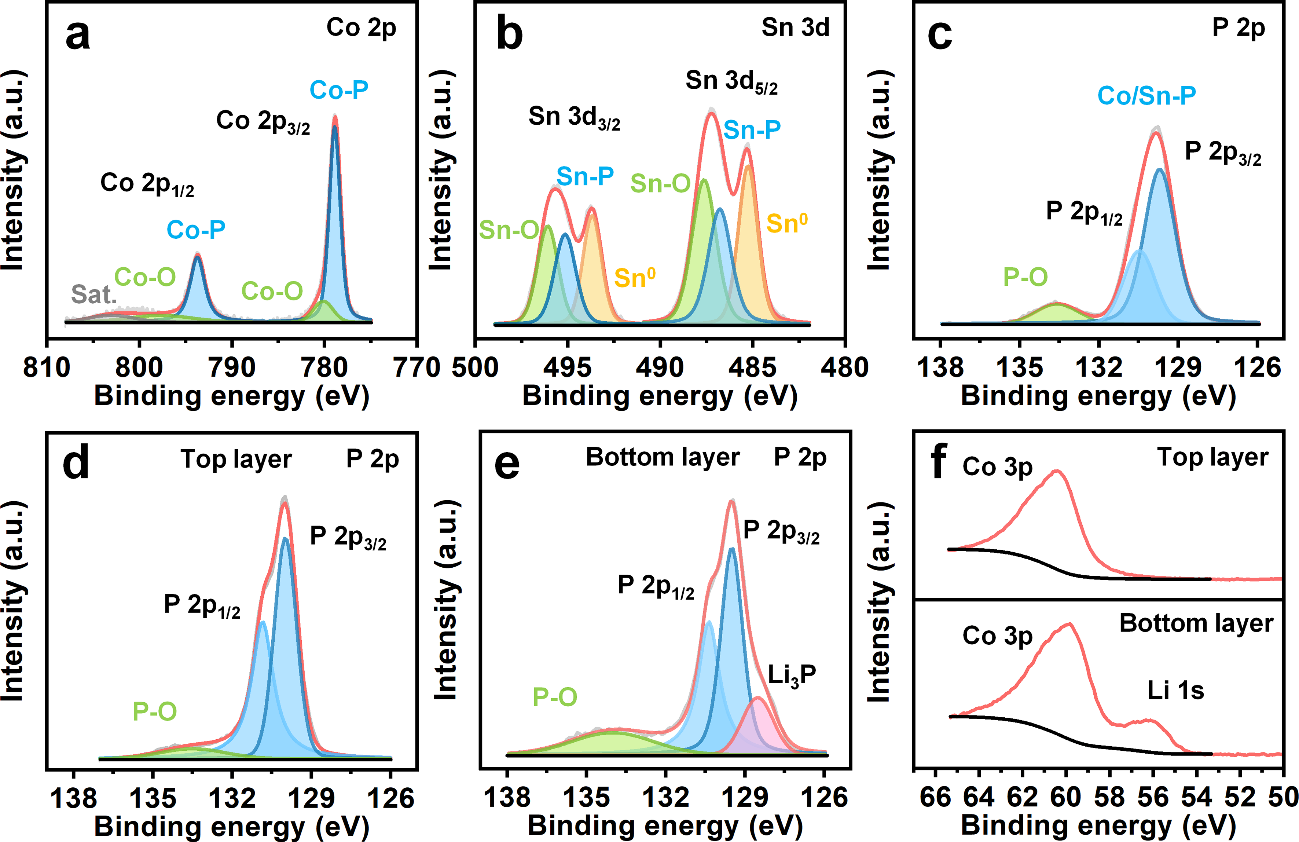


**Fig. S34** High resolution XPS spectra of pristine SCP@Li before Ar^+^ etching. **a** Co 2p, **b** Sn 3d, and **c** P 2p. P 2p XPS spectra of **d** SCP@Li and **e** SCP@Li with thinner layer after Ar^+^ etching. **f** Li 1s XPS spectra of SCP@Li and SCP@Li with thinner layer after Ar^+^ etching


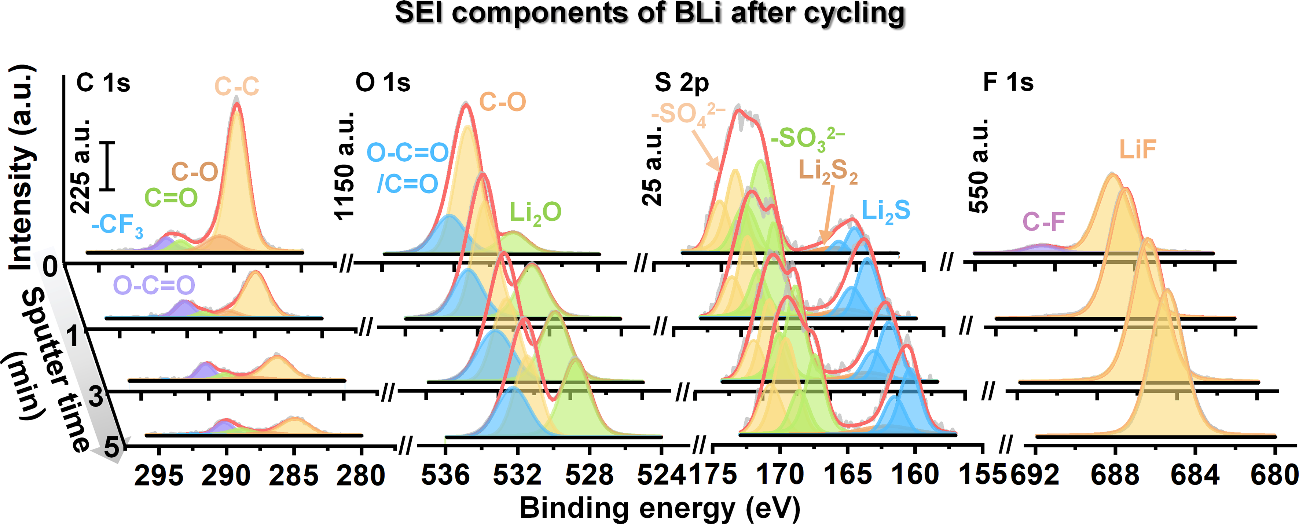


**Fig. S35** Depth-profiled XPS spectra of SEI layer on BLi after cycling


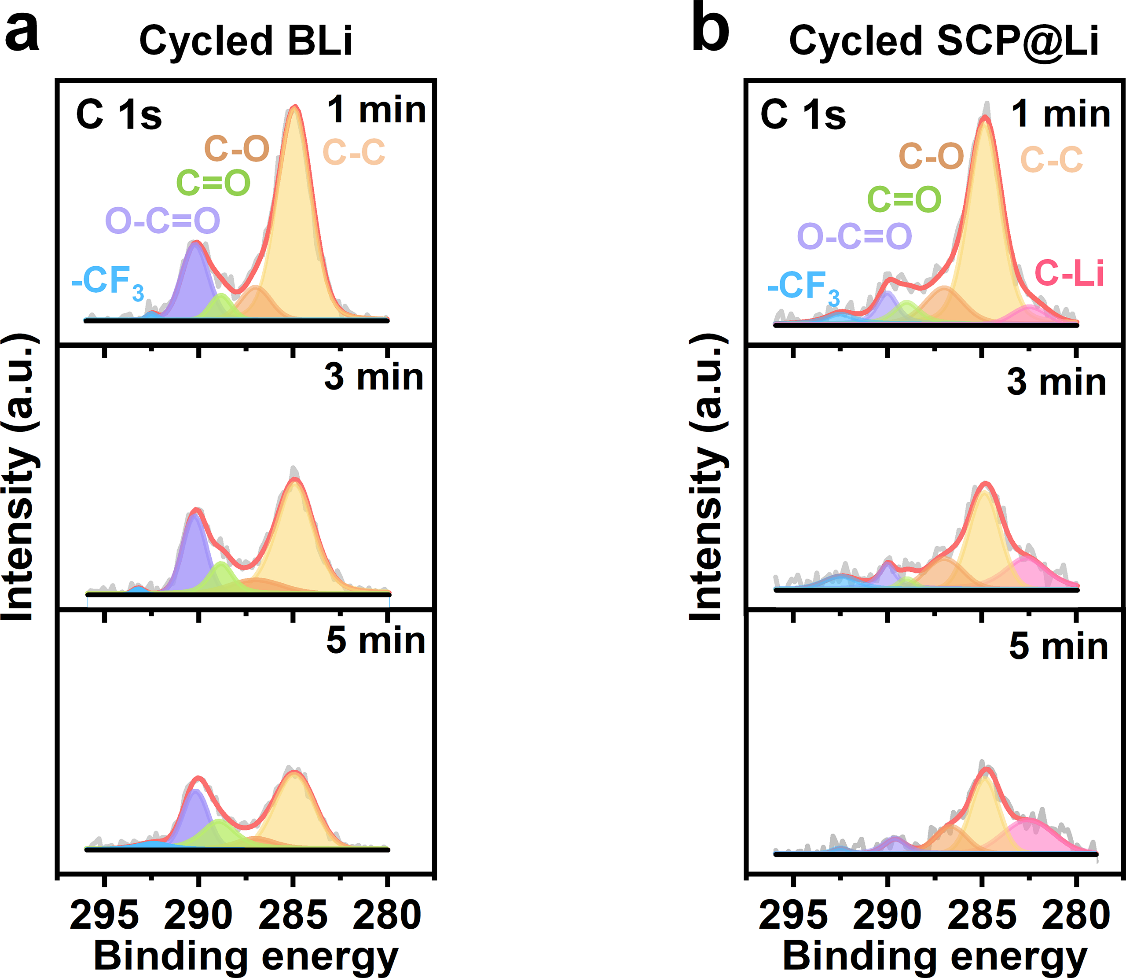


**Fig. S36** C 1s XPS spectra of **a** cycled BLi and **b** SCP@Li at different Ar^+^ sputtering times


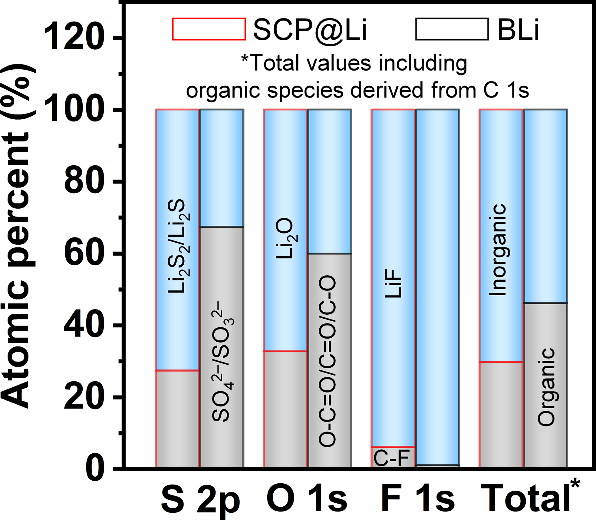


**Fig. S37** Atomic proportions of SEIs on SCP@Li and BLi calculated from depth-profiled XPS spectra (5 min)


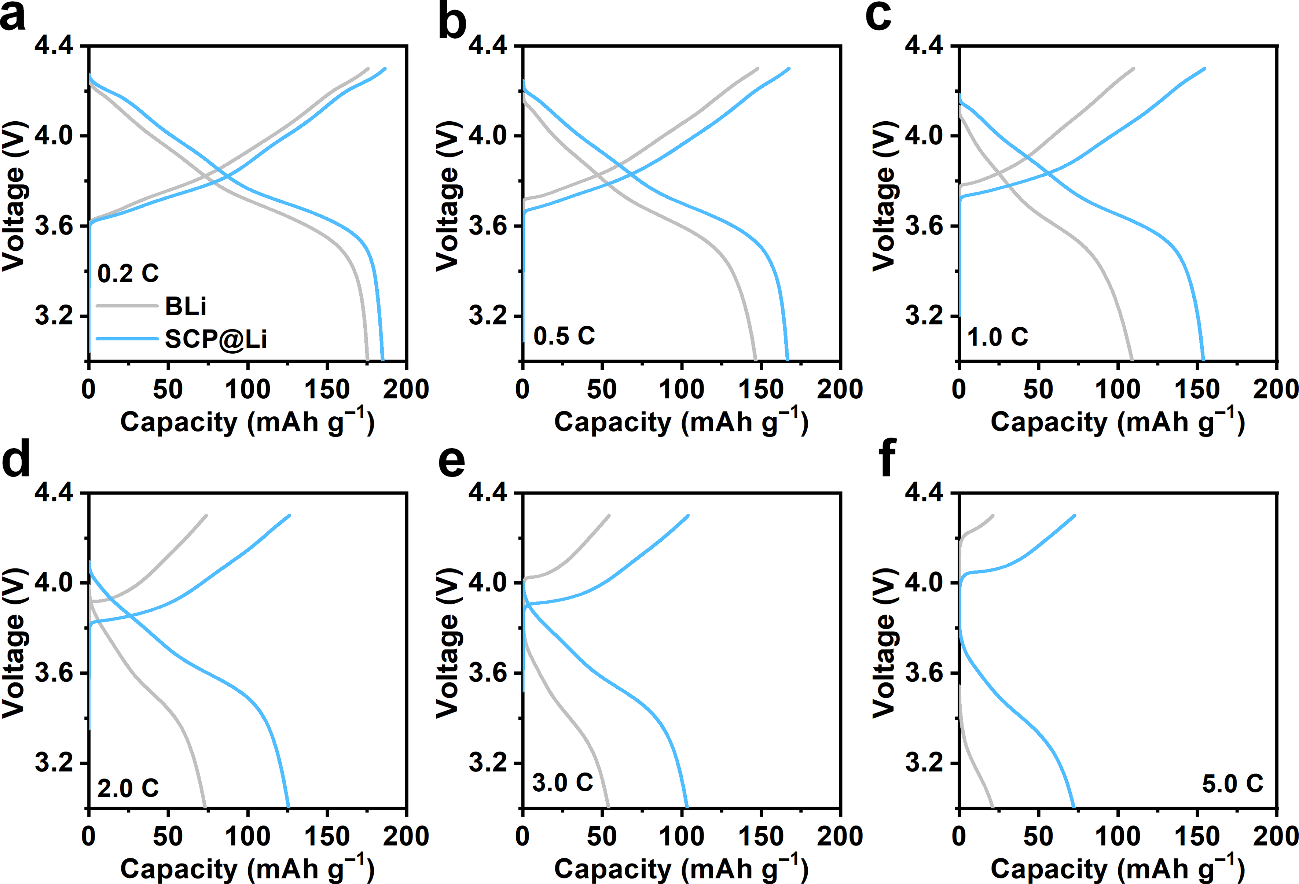


**Fig. S38** Galvanostatic profiles of NCM811//SCP@Li and NCM811//BLi cells at various C-rates. **a** 0.2 C, **b** 0.5 C, **c** 1.0 C, **d** 2.0 C, **e** 3.0 C, and **f** 5.0 C

**Table S1** Energy positions of $\text{ϕ}_{\text{Wf}}$, E_VBM_, and E_g_ of CoP and SnP_0.94_

| Energy  levels  Samples | $\text{ϕ}_{\text{Wf}}$ | **E_VBM_** | **E_g_** |
| --- | --- | --- | --- |
| **CoP** | 4.2 eV | 0.25 eV | 1.25 eV |
| **SnP_0.94_** | 3.17 eV | 1.16 eV | 1.45 eV |

**Table S2** Comparison of electrochemical resistances in BLi and SCP symmetric cells before/after cycling (R_int_ = R_SEI_ + R_ct_)

| Resistances  Samples | **R_SEI_** | **R_ct_** | **R_int_** |
| --- | --- | --- | --- |
| **BLi before cycling** | 12.3 Ω | 295.1 Ω | 307.4 Ω |
| **BLi after cycling** | 5.1 Ω | 48.6 Ω | 53.7 Ω |
| **SCP@Li before cycling** | 1.1 Ω | 1.9 Ω | 3.0 Ω |
| **SCP@Li after cycling** | 0.54 Ω | 1.0 Ω | 1.54 Ω |

**Table S3** Comparison of the performance of symmetric cells using previously reported surface-modified Li metal anodes and SCP@Li

| **Composition** | **Current density (mA cm^–2^)** | **Areal capacity (mAh cm^–2^)** | **Over**  **potential**  **(mV)** | **Cycle**  **(No.)** | **References** |
| --- | --- | --- | --- | --- | --- |
| **SnP_0.94_/CoP** | **1** | **1** | **14.5** | **600** | **This work** |
|  | **5** |  | **68** | **750** |  |
| BF_3_-doped MXene | 1 | 1 | 33.3 | 500 | [S1] |
| Ag@C_3_N_4_ | 1 | 1 | 28 | 700 | [S2] |
| Ag@C | 1 | 1 | 13 | 500 | [S3] |
| Zn_3_Si_4_O_10_(OH)_2_ | 1 | 1 | 30 | 425 | [S4] |
|  | 2 |  | 50 | 750 |  |
| MoS_2_@C | 0.5 | 1 | 15 | 250 | [S5] |
|  | 1 |  | 22 | 375 |  |
| Montmorillonite | 0.5 | 0.5 | 10 | 500 | [S6] |
|  | 3 | 3 | 80 | 35 |  |
| Ag@CuO | 1 | 1 | 25 | 525 | [S7] |
| SbCl_3_ | 1 | 1 | 20 | 200 | [S8] |
| LiF-Li_3_N | 0.5 | 0.5 | 50 | 500 | [S9] |
| Cetyltrimethylammonium bromide | 0.5 | 1 | 50 | 325 | [S10] |

**Table S4** Comparison of specific capacities of LFP//BLi and LFP//SCP@Li cells at different C-rates

| C-rate  Cells | **0.2 C** | **0.5 C** | **1.0 C** | **2.0 C** | **3.0 C** | **5.0 C** | **10 C** | **0.5 C**  **(recover)** |
| --- | --- | --- | --- | --- | --- | --- | --- | --- |
| **LFP//BLi** | 151.0 | 140.8 | 127.2 | 105.2 | 88.3 | 65.9 | 36.9 | 136.7 |
| **LFP//SCP@Li** | 157.2 | 150.5 | 140.9 | 125.9 | 113.6 | 95.6 | 68.5 | 148.6 |

**Supplementary References**

1. M. Shang, O.G. Shovon, F.E.Y. Wong, J. Niu, A BF_3_-Doped MXene dual-layer interphase for a reliable lithium-metal anode. Adv. Mater. **35**(8), 2210111 (2023). <https://doi.org/10.1002/adma.202210111>
2. H. Lim, M. Choi, H. Kang, W. Choi, Accelerating lithium deposition kinetics *via* lithiophilic Ag-decorated graphitic carbon nitride spheres for stable lithium metal anode. Energy Environ. Mater. **8**(2), e12830 (2025). <https://doi.org/10.1002/eem2.12830>
3. Y. Fang, S.L. Zhang, Z.-P. Wu, D. Luan, X.W.D. Lou, A highly stable lithium metal anode enabled by Ag nanoparticle–embedded nitrogen-doped carbon macroporous fibers. Sci. Adv. **7**(21), eabg3626 (2021). <https://doi.org/10.1126/sciadv.abg3626>
4. H.-J. Liu, C.-Y. Yang, M.-C. Han, C.-Y. Yu, X. Li et al., *In-situ* constructing a heterogeneous layer on lithium metal anodes for dendrite-free lithium deposition and high Li-ion flux. Angew. Chem. **135**(11), e202217458 (2023). <https://doi.org/10.1002/ange.202217458>
5. L. Yu, Q. Su, B. Li, L. Huang, G. Du et al., Pre-lithiated Edge-enriched MoS2 nanoplates embedded into carbon nanofibers as protective layers to stabilize Li metal anodes. Chem. Eng. J. 2022, 429, 132479. <https://doi.org/10.1016/j.cej.2021.132479>
6. Y. Nan, S. Li, C. Han, H. Yan, Y. Ma et al., Interlamellar lithium-ion conductor reformed interface for high performance lithium metal anode. Adv. Funct. Mater. **31**(25), 2102336 (2021). <https://doi.org/10.1002/adfm.202102336>
7. M. Gao, Q. Dong, M. Yao, X. Wang, J. Li et al., Dual-gradient engineering of urchin-like silver@copper oxide heterostructures for highly stable lithium metal anodes. Adv. Funct. Mater. **34**(28), 2401442 (2024). <https://doi.org/10.1002/adfm.202401442>
8. B.C. Min, J.B. Park, C. Choi, D.-W. Kim, Dynamic construction of a composite solid electrolyte interphase for dendrite-free lithium metal batteries *via* lithium-antimony self-alloying. Adv. Compos. Hybrid Mater. **8**, 4 (2025). <https://doi.org/10.1007/s42114-024-01070-7>
9. K. Zeng, Q. Liu, H. Ma, G. Zhao, Q. An, In situ co-growth LiF-Li_3_N rich dual-protective layers enable high interface stability for solid-state lithium-metal batteries. Energy Storage Mater. **70**, 103564 (2024). <https://doi.org/10.1016/j.ensm.2024.103564>
10. Z. Sun, J. Yang, H. Xu, C. Jiang, Y. Niu et al., Enabling an inorganic-rich interface via cationic surfactant for high-performance lithium metal batteries. Nano-Micro Lett. **16**(1), 141 (2024). <https://doi.org/10.1007/s40820-024-01364-x>
